# Rational design and synthesis of 2-(1*H*-indazol-6-yl)-1*H*-benzo[d]imidazole derivatives as inhibitors targeting FMS-like tyrosine kinase 3 (FLT3) and its mutants

**DOI:** 10.1080/14756366.2021.2020772

**Published:** 2022-01-23

**Authors:** Daseul Im, Joonhong Jun, Jihyun Baek, Haejin Kim, Dahyun Kang, Hyunah Bae, Hyunwook Cho, Jung-Mi Hah

**Affiliations:** a Department of Pharmacy, College of Pharmacy, Hanyang University, Ansan, Korea; b Institute of Pharmaceutical Science and Technology, Center for Proteinopathy, Hanyang University, Ansan, Korea

**Keywords:** FLT3, FLT3-ITD, FLT3-D835Y, Benzimidazole, Indazole

## Abstract

Fms-like tyrosine kinase 3 (FLT3) has been verified as a therapeutic target for acute myeloid leukaemia (AML). In this study, we report a series of 2-(1*H*-indazol-6-yl)-1*H*-benzo[d]imidazol-5-yl benzamide and phenyl urea derivatives as potent FLT3 inhibitors based on the structural optimisation of previous FLT3 inhibitors. Derivatives were synthesised as benzamide **8a**–**k**, **8n**–**z**, and phenyl urea **8l**–**m**, with various substituents. The most potent inhibitor, **8r**, demonstrated strong inhibitory activity against FLT3 and FLT3 mutants with a nanomolar IC_50_ and high selectivity profiles over 42 protein kinases. In addition, these type II FLT3 inhibitors were more potent against FLT3 mutants correlated with drug resistance. Overall, we provide a theoretical basis for the structural optimisation of novel benzimidazole analogues to develop strong inhibitors against FLT3 mutants for AML therapeutics.

## 1. Introduction

Acute myeloid leukaemia, one of the most notorious types of leukaemia, is characterised by the abnormal proliferation and accumulation of immature cells in bone marrow and peripheral tissues. Consequently, it leads to lack of normal haematopoietic cells, leading to the characteristic symptoms of AML, such as fatigue, anaemia, dyspnoea, bleeding, and severe infections due to poorly differentiated progenitor cells^1^**^,^**^2^. According to the American Cancer Society’s estimates for AML in 2020, newly diagnosed cases of AML and deaths from AML are as high as 19,940 and 11,180 in the United States, respectively. It is reported that AML incidence rates generally increase alongside age, and the five-year overall survival rate for patients with AML is less than 50%^3^**^,^**^4^. Recent genomic sequencing analysis reported that mutations in FLT3 commonly discovered, and FLT3 has been considered a potential therapeutic target against AML^5^.

FLT3, classified as a type of trans-membrane receptor tyrosine kinase, is expressed on lympho-haematopoietic cells. When Fms-related tyrosine kinase 3 ligand (FLT3 ligand) binds to receptor tyrosine kinase (RTK), FLT3 is activated by dimerisation and auto-phosphorylation of kinase domain, which activates its series of downstream signalling pathways, including Jasnus kinase/signal transducer and activator of transcription (JAK/STAT), Ras/mitogen activated protein kinase (RAS/MAPK), and phosphatidylinositol-3-kinase/AKT/mammalian target of rapamycin (PI3K/AKT/mTOR) pathways mediating the proliferation, survival, and immune response of haematopoietic stem cells and progenitor cells^6–9^.

**Figure 1.**
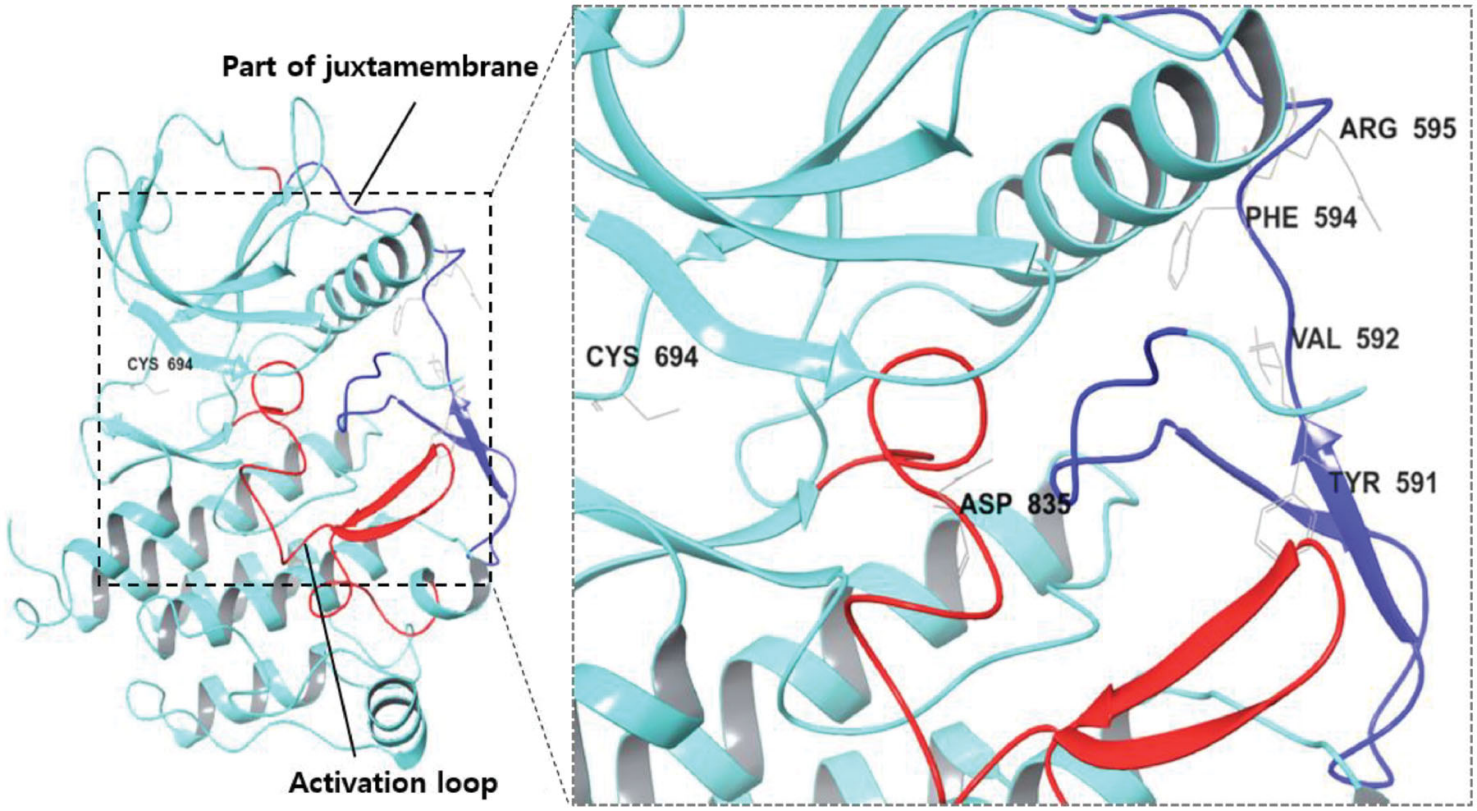
(Left) X-ray crystallography of FLT3 kinase (PDB:4RT7); (Right) The region and amino acid residues in which FLT3 mutations commonly occur; (Blue) Juxtamembrane; (Red) Activation loop of the kinase domain.

But mutation in FLT3 can lead to its auto-phosphorylation and activation without FLT3 ligand binding. FLT3 internal tandem duplication (FLT3-ITD) occurs in the juxtamembrane domain (JMD) as a form of sequence replication and was found in 20–30% of AML patients; it is primarily related to poor AML prognosis. The FLT3-ITD insertion mutation is frequently observed between Tyr591 and Val592 or Phe594 and Arg595. FLT3 point mutation in the tyrosine kinase domain (TKD) is present in 5% of AML patients, and mutations at Asp835 which most commonly occur are considered to be a part of the AML drug resistance mechanism^10–13^ (Figure 1).

**Figure 2.**
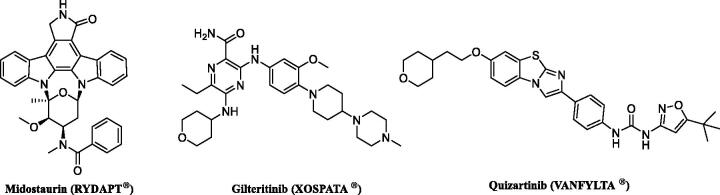
FLT3 inhibitors approved for AML treatment.

On the basis of their binding mode, all known FLT3 inhibitors can be classified as type I or type II. Type I inhibitors are competitive inhibitors that can bind with the active conformation of FLT3 (DFG-in conformation). Sunitinib, midostaurin, lestaurtinib, crenolanib, and gilteritinib have been reported as type I FLT3 inhibitors that tightly bind with its active conformation. But type I inhibitors lack selectivity for FLT3 and also show strong affinity for other kinases due to high similarities in ATP binding sites. Type II inhibitors interact with the DFG-out conformation, the inactive conformation, and also bind with an additional hydrophobic site adjacent to the ATP-binding pocket^14–16^. Type II inhibitors generally possess higher selectivity for target kinases because hydrophobic pockets are less conserved regions compared to ATP binding sites^17^. Sorafenib and quizartinib have been reported as type II FLT3 inhibitors. Among the mentioned FLT3 inhibitors, only two molecules, including midostaurin (Rydapt^®^) and gilteritinib (Xospata^®^), have been approved by the FDA for the treatment of FLT3-mutated AML in 2017 and 2018, respectively. Quizartinib (VANFLYTA^®^ in Japan) has received regulatory approval in Japan for use in FLT3-ITD-positive patients in 2019^18^^,^^19^ (Figure 2).

**Figure 3.**
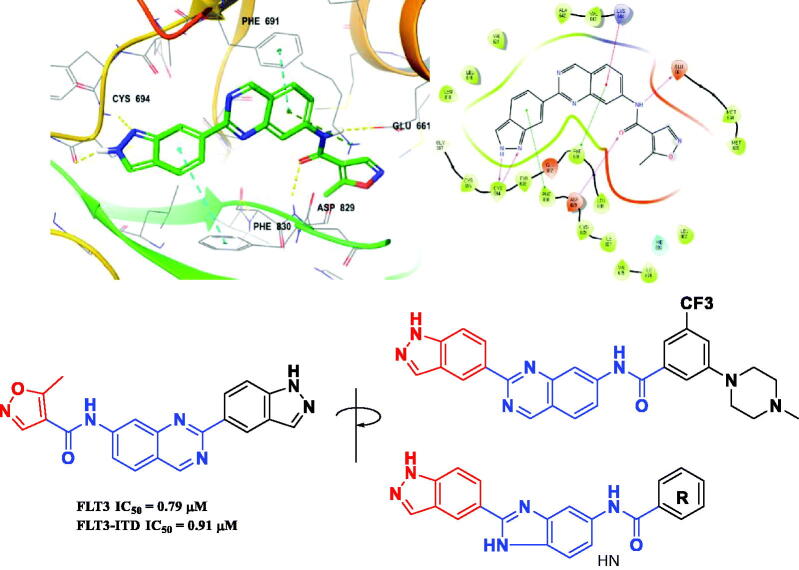
Docking structures of a quinazoline derivative with an indazole fragment in FLT3 (PDB: 4RT7) and Design of a benzimidazole analog with an indazole moiety from studies on the binding mode of a previous FLT3 inhibitor.

In our previous study, benzimidazole and quinazoline derivatives were synthesised and optimised from FMS inhibitors as selective type II FLT3 inhibitors^20^^,^^21^. We found that core structures including benzimidazole and quinazoline play a key role in retaining their binding mode that interacts with the Phe691 residue in FLT3 kinase *via* π-π interaction by means of investigating molecular docking. On the process of structure optimisation to enhance activity, it was verified that indazole fragments could be employed as a hinge binder that binds with the ATP-binding pocket^21^ (Figure 3). Based on this observation, we replaced the isoxazole structure with various phenyl amide groups retaining its quinazoline core structure first and synthesised several derivatives, including 5-methylisoxazole-4-carboxamide and 3–(4-methylpiperazin-1-yl)-5-(trifluoromethyl)benzamide, which were considered as starting points to develop novel FLT3 inhibitors. We then decided to modify the quinazoline structure for further optimisation to improve its handling properties and the flexibility of its structure. As a result, we introduced a benzimidazole scaffold instead of quinazoline as a bioisostere with chemically and biologically similar properties to quinazoline at the binding site of the FLT3^22^. Finally, we diversified its R group seeking a substituent to fit with the hydrophobic pocket adjacent to the FLT3 active site, resulting in the synthesis of novel benzimidazole derivatives containing an indazole fragment (**8a–z**) (Figure 3).

## 2. Results and discussion

### 2.1. Chemistry

**Scheme 1.**
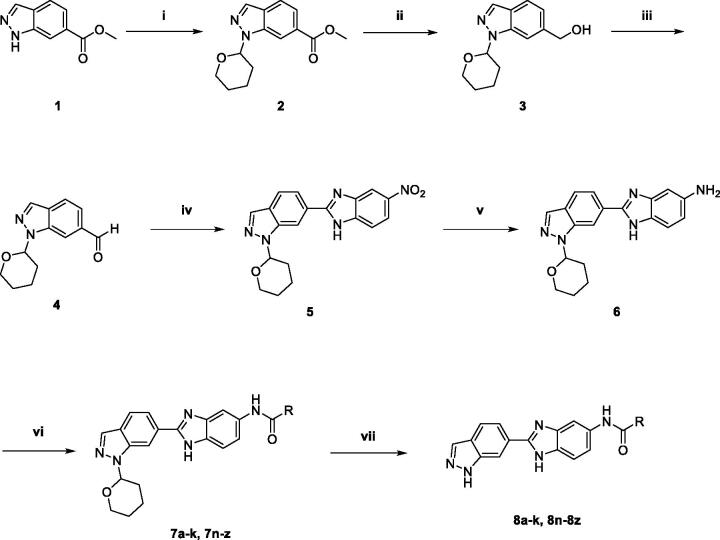
Synthesis of *N*-(2-(1*H*-indazol-6-yl)-1*H*-benzo[d]imidazol-5-yl)benzamide derivatives. (i) 3,4-dihydro-2*H*-pyran, Pyridinium *p*-toluenesulfonate, µW, 50 °C, 5 h; (ii) LiAlH_4_ in THF, THF, 0 °C; (iii) Dess-Martin periodinane, MC/THF = 1:1, rt; (iv) 4-Nitrobenzene-1,2-diamine, NH_4_Cl, EtOH, reflux; (v) H_2_, Pd/C, EtOH; (vi) Benzoic acid, EDC, HOBt, TEA, THF, rt; (vii) 20% TFA, CH_2_Cl_2_ or 5% HCl in EtOH.

**Scheme 2.**
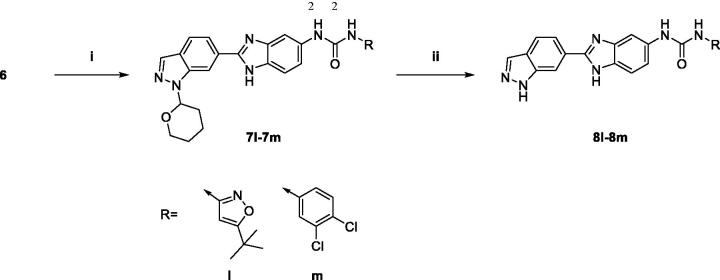
Synthesis of 1–(2-(1*H*-indazol-6-yl)-1*H*-benzo[d]imidazol-5-yl)-3-phenylurea derivatives. (i) (1) 4-Nitrophenyl chloroformate, DIPEA, THF, 0 °C; (2) RNH_2_, THF, 50 °C; (ii) 20% TFA, CH_2_Cl_2_.

The general synthetic route for benzimidazole analogues is shown in Scheme 1. The synthesis of benzimidazole derivatives started from commercially available methyl 1*H*-indazole-6-carboxylate (**1**). Methyl 1*H*-indazole-6-carboxylate (**1**) was treated with 3,4-dihydro-2*H*-pyran (DHP) under mild acidic conditions and subjected to microwave irradiation at 50 °C to introduce a DHP group and yield a protected compound (**2**)^23^. Methyl 1-(tetrahydro-2*H*-pyran-2-yl)-1*H*-indazole-6-carboxylate (**2**) was reduced to an alcohol (**3**) using LiAlH_4_ and oxidised to an aldehyde compound (**4**) with Dess-Martin Periodinane. Aldehyde (**4**) was treated with NH_4_Cl and 4-nitrobenzene-1, 2-diamine to obtain a benzimidazole compound (**5**) as a core intermediate^24^. Subsequently, nitro group of benzimidazole (**5**) was reduced to amino group under H_2_. Aniline (**6**) was coupled with various benzoic acids, and then deprotection was performed under acidic conditions to produce the final benzimidazole derivatives (**8a**–**z**). Also, aniline (**6**) was coupled with an amine *via* urea coupling, and deprotection was performed to obtain the final benzimidazole derivatives (**8l**–**m**) (Scheme 2).

### 2.2. In vitro *structure-activity relationship (SAR) studies and structural modification*

All benzimidazole compounds, **8a**–**z**, were evaluated for activity against FLT3 and FLT3-D835Y, and the results are shown in Table 1. While most compounds exhibited potent and selective activity with FLT3 kinase, they were especially more potent against FLT3-D835Y, which is mainly related to the mechanism of secondary resistance to FLT3 inhibitors^25^. Compound **8r** with an ethyl piperazine moiety showed the most potent activity with FLT3, with an IC_50_ value of 41.6 nM, and FLT3-D835Y, with an IC_50_ value of 5.64 nM. Structure-activity relationships (SARs) were inferred from activity data.

**Table 1.** Enzymatic activities of *N*-(2-(1*H*-indazol-6-yl)-1*H*-benzo[d]imidazol-5-yl)benzamide and 1–(2-(1*H*-indazol-6-yl)-1*H*-benzo[d]imidazol-5-yl)-3-phenylurea derivatives

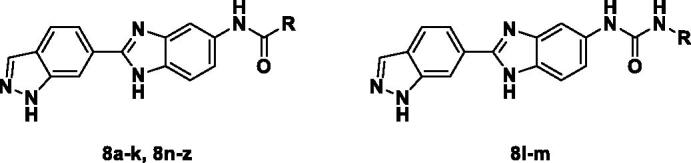							
No.	R	FLT3	FLT3 (D835Y)	No.	R	FLT3	FLT3 (D835Y)
		IC_50_ (µM)				IC_50_ (µM)	
**8a**	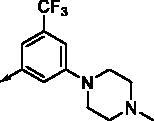	0.181	0.021	**8n**	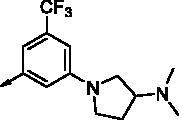	0.287	0.113
**8b**	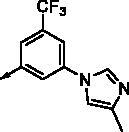	0.639	0.110	**8o**	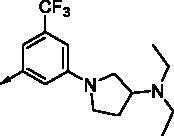	0.841	0.120
**8c**	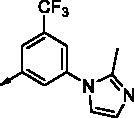	0.470	0.108	**8p**	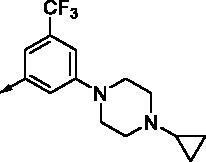	0.409	0.131
**8d**	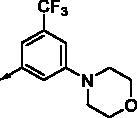	1.03	0.151	**8q**	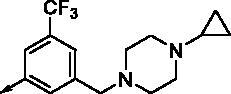	0.525	0.174
**8e**	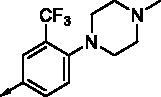	0.154	0.044	**8r**	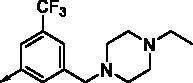	0.0416	0.006
**8f**	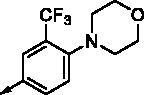	2.51	0.370	**8s**	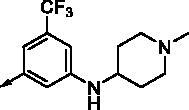	0.158	0.025
**8g**	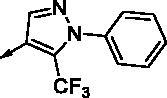	1.10	0.063	**8t**	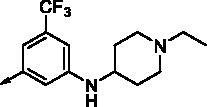	0.285	0.087
**8h**	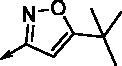	0.702	0.082	**8u**	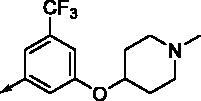	0.289	0.055
**8i**	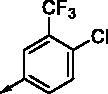	0.676	0.084	**8v**	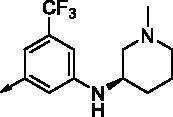	0.107	0.009
**8j**	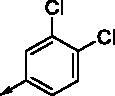	0.514	0.103	**8w**	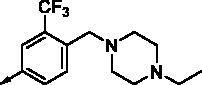	0.29	0.013
**8k**	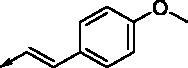	0.761	0.191	**8x**	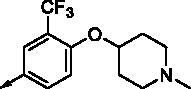	0.743	0.145
**8l**	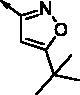	1.17	0.261	**8y**	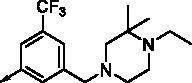	0.145	0.042
**8m**	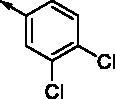	3.58	1.010	**8z**	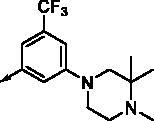	0.066	0.046
**Staurosporine**						1.82 nM	0.073 nM

**Figure 4.**
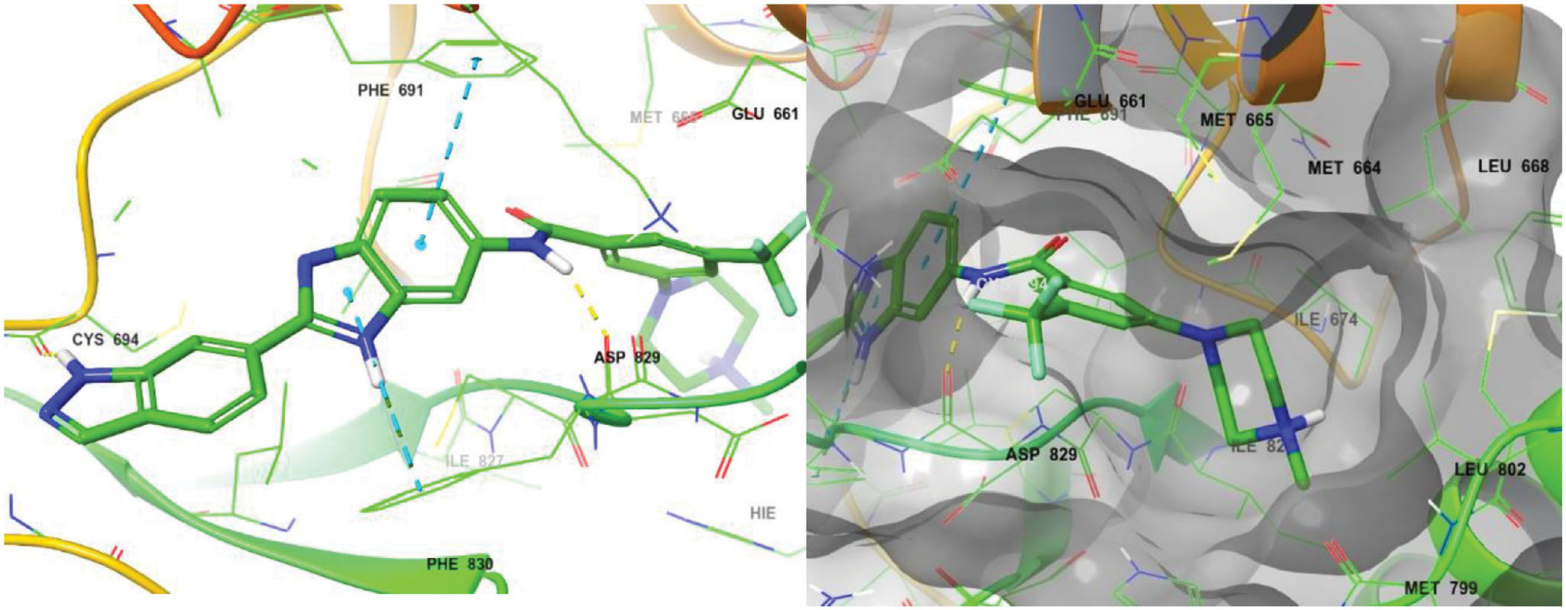
Docking structure of compound **8a** at the active site of FLT3 (Left) and at the hydrophobic pocket adjacent to the ATP-binding site (Right).

In previous work, we designed and synthesised quinazoline compounds incorporating indazole. Through investigations of molecular docking, we observed that the indazole structure plays a key role as a hinge binder in type II inhibitors to interact with the Cys694 residue in FLT3 (Figure 3). We introduced an indazole moiety as a hinge binder and replaced the quinazoline core structure with benzimidazole (Figure 3). Most benzimidazole derivatives retained their activity and showed enhanced potency against FLT3 (**8a**, **8b**, **8d**, **8f**, **8g**, **8h**, **8i**, **8k**). Especially, compounds **8b** and **8d** were about 2- to 4-fold more potent (IC_50_ values of 0.639 µM and 1.03 µM, respectively) compared to the corresponding quinazoline series (IC_50_ values of 1.58 µM and 3.98 µM, respectively). Among those compounds, compounds **8a** and **8e** incorporating methyl piperazine were the most potent, exhibiting IC_50_ values of 0.181 and 0.154 µM, respectively, against FLT3. The newly synthesised FLT3 inhibitors nicely bound to the active site of FLT3 via hydrogen bonding and π-π interaction. The N-H of the indazole and N-H of the amide group substituted in the benzimidazole form hydrogen bonds with the amide backbones of Cys694 and Asp829 in FLT3. The fused ring system of benzimidazole interacts with the phenyl side chains of Phe691 and Phe830. These interactions of core structure could lead to the development of FLT3 inhibitors with retained activity and further improved potency (Figure 4).

Following the introduction of indazole and benzimidazole, we modified the inhibitor’s structures to optimise their activity by exploiting linkage connecting their fragments and the pocket adjacent to the ATP-binding site. This pocket then had more space which could be occupied by FLT3 inhibitors, and the terminal region of the pocket is surrounded by hydrophobic residues, such as Met664, Met665, Leu668, Ile674, Met799, Leu802, and Ile827 (Figure 4).

Compounds **8l** and **8m** were synthesised to replace the amide to urea linkage between the benzimidazole core structure and the phenyl group. But its potency was about 2- to 7-fold less than that of **8h** and **8j**. Then we substituted methyl imidazole (**8b**, **8c**) with *N*,*N*-dimethyl and diethyl pyrrolidine moieties, respectively, to elongate their terminal structure. Compound **8n** displayed a 3-fold more potent activity than that of **8o**. The *N*-methyl group might be the proper substituent size to occupy the hydrophobic pocket next to the ATP-binding site.

For further structural optimisation, we modified the scaffold of compound **8a**, which displayed the most potent activity towards FLT3 among **8a**–**o**, and replaced its methyl substituent with a cyclopropyl group (**8p**). Although compound **8p** retained potency against FLT3, improved activity was not achieved. Next, we added one atom, such as C, N, or O, between the phenyl group and basic amine substituent to elongate the scaffold length and give its basic amine substituent flexibility (**8q**–**v**). As a result, compounds **8r**, **8s**, and **8v** demonstrated improved activity. Analogues **8r** and **8v** showed about 2- to 4-fold more potent activities against FLT3. Furthermore, these compounds have single-digit nanomolar FLT3-D835Y inhibitory activities.

Benzimidazole derivatives with 1, 3, 4-substituted phenyl groups (**8w**, **8x**) were about 3- to 7-fold less potent than those with 1, 3, 5-substituted phenyl groups (**8r**, **8u**). Although **8a** and **8e** showed only similar activity according to the orientation of substitution, compounds **8w** and **8x**, despite their optimised structures, displayed quite decreased activity compared with compounds **8r** and **8u**. It is implied that a 1, 3, 5-substituted phenyl group occupies the hydrophobic pocket at the binding site of FLT3 kinase.

In order to optimise its terminal, basic substituent, such as piperazine, a dimethyl group was incorporated (**8y**, **8z**). Analogue **8y** had less potent activity compared to **8r**, but compound **8z** had a 3-fold more potent activity towards FLT3 (IC_50_ value of 65.9 nM) than **8a**.

### 2.3. Enzymatic inhibitory activities against wild-type FLT3 and activated FLT3 mutants

The most potent compound, **8r**, was examined further for inhibitory activity against FLT3 mutants, and the results are shown in Table 2. **8r** displayed a similar or higher level of potency towards FLT3-ITD-NPOS and W51 (IC_50_ = 41.5 nM, 22.8 nM, respectively) compared with that towards wild-type FLT3 (IC_50_ = 41.6 nM). FLT3-ITD mutations mainly occur in the juxtamembrane domain, which is away from the active site of FLT3, so FLT3 inhibitors generally exhibit similar potency against FLT3-ITD mutants^25^. But FLT3-TKD mutants like D835Y occur in the active site of the kinase; Point mutation in this region could induce a constitutively active conformation of FLT3, and that’s why some type II kinase inhibitors which bind with the inactive form of kinase could generally not bind well enough to such FLT3 mutants^26^. This is known as a mechanism of secondary resistance to type II FLT3 inhibitors^25^. However, our benzimidazole derivatives designed as type II inhibitors had a tendency to show more potent activity against FLT3-TKD mutants like FLT3-D835Y. Especially, compounds **8r** and **8v** were about 7- to 12-fold more potent against FLT3-D835Y (IC_50_ = 5.64 nM, 8.86 nM, respectively) than against wild-type FLT3 (IC_50_ = 41.6 nM, 107 nM, respectively). It is impressive result to suggest a potential that might avoid secondary resistance mechanism^12^^,^^13^^,^^25^^,^^27^. Additionally, compound **8r** also displayed similar or more potent activities against other FLT3-TKD mutants, such as FLT3 (F594_R595 ins R), FLT3 (F594_R595 ins REY), FLT3 (R595_E596 ins EY), and FLT3 (Y591_V592 ins VDFREYEYD) compared with wild-type FLT3 (Table 2).

**Table 2.** Enzymatic inhibitory activities of compound **8r** against FLT3 mutants.

Kinase	IC_50_
FLT3 wild type	41.6 nM
FLT3 (ITD)-NPOS	41.5 nM
FLT3 (ITD)-W51	22.8 nM
FLT3 (D835Y)	5.64 nM
FLT3 (F594_R595 ins R)	37.3 nM
FLT3 (F594_R595 ins REY)	41.6 nM
FLT3 (R595_E596 ins EY)	24.8 nM
FLT3 (Y591_V592 ins VDFREYEYD)	49.5 nM

### 2.4. Protein kinase profiling assay

**Figure 5.**
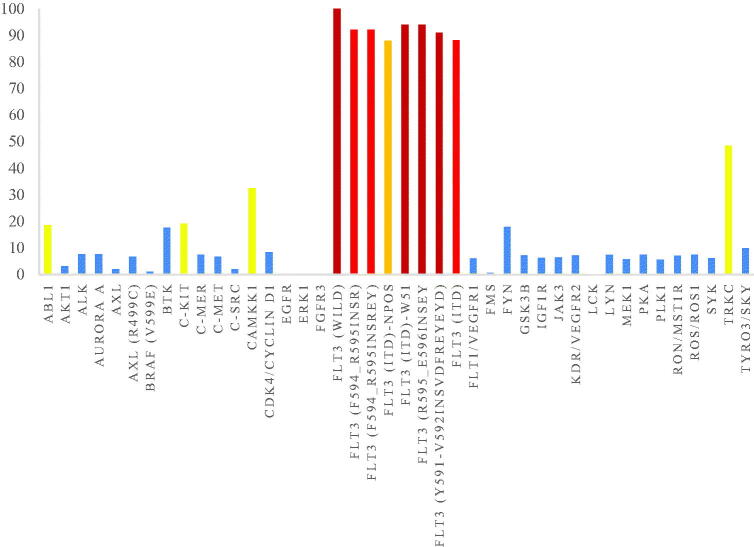
The profiles of the compound **8r** (1 µM) are shown.

As shown in Figure 5, the representative compound **8r** was tested against 42 different kinases at a single dose concentration of 1 µM. It was revealed that compound **8r** showed an excellent selectivity profile. **8r** had strong inhibitory activity against the FLT3-ITD mutant significantly related to the therapeutic target of AML, having the activity level of inhibition below 20% on most of the other kinases. While this compound displayed strong inhibitory activities on other FLT3 mutants including FLT3 (F594_R595insR), FLT3 (F594_R595insREY), FLT3 (ITD)-NPOS, FLT3 (ITD)-W51, FLT3 (R595_E596insEY) and FLT3 (Y591_V592insVDFREYEYD), compound **8r** effectively inhibited CAMKK1, and TRKC kinase activities with an activity level of inhibition over 20%. We also evaluated the enzymatic inhibition of compound **8r** towards ABL1, c-Kit, CAMKK1, and TRKC (Table 3). Compound **8r** demonstrated excellent activity against wild-type FLT3 and FLT3 mutants (a nanomolar level of IC_50_ shown in Table 2), but only good potency against CAMKK1 and TRKC (IC_50_ = 0.395 µM, and 1.52 µM, respectively).

**Table 3.** IC_50_ for the enzymatic inhibitory activity of compound **8r**.

	IC_50_			
	ABL1	c-Kit	CAMKK1	TRKC
**8r**	3.09 µM	4.57 µM	0.395 µM	1.52 µM

### 2.5. Molecular docking studies

**Figure 6.**
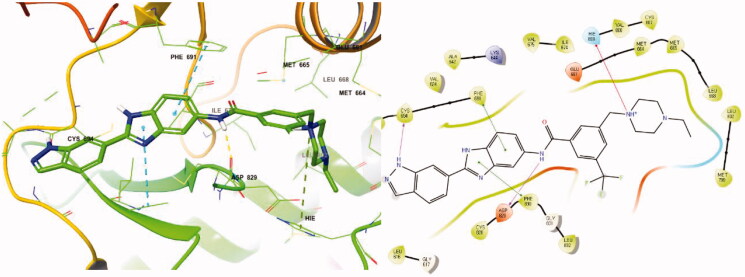
Docking structure of **8r** in FLT3 (PDB: 4RT7)^26^.

Molecular docking of compound **8r** into the ATP binding site of FLT3 (PDB:4RT7) was analysed utilising Maestro v12.7 (Scrhödinger Release 2021–1) to better understand the interactions between benzimidazole derivatives and FLT3 kinase. The docking result is described in Figure 6. The amino hydrogen atom of the **8r** indazole forms a hydrogen bond (N-H/O: 1.95 Å) with the amide oxygen atom of Cys694 and another hydrogen bond (1.90 Å) between the N-H of the **8r** amide bond and the oxygen atom of Asp829, which suggests that the indazole group plays an important role in the receptor-ligand complex. The benzimidazole fused ring of **8r** forms a π-π interaction with the phenyl groups of Phe691 and Phe830. These interactions also perform a significant role in FLT3 kinase inhibition due to its conserved residue Phe691. On the other hand, FMS kinase has a Thr663 residue in the same sequence at the active site. Therefore, compound **8r** could not form a π-π interaction with Thr663, which explains its selective inhibition between FLT3 and FMS kinase^21^. The positively charged nitrogen of the ethyl piperazine ring forms a π-cation interaction with His809. Furthermore, ethyl moiety substituted in the piperazine occupies the terminal hydrophobic pocket of the FLT3 active site surrounded by hydrophobic amino acid residues. It might enhance the binding affinity, possibly resulting in increased inhibition of kinase activity. The light blue colour region in Figure 6 shows the hydrophobic regions around the ethyl substituent.

## 3. Conclusions

A series of *N*-(2-(1*H*-indazol-6-yl)-1*H*-benzo[d]imidazol-5-yl)benzamide derivatives incorporating various aromatic substituents were synthesised by modifying quinazoline derivatives reported as novel FLT3 inhibitors that exhibited activities against FLT3, FLT3-ITD mutants, and FLT3-TKD mutants. Compound **8r** displayed the most potent inhibitory activity against FLT3 with an IC_50_ of 41.6 nM and was also active against FLT3-ITD (W51) and FLT3-TKD (D835Y) with IC_50_ values of 22.8 nM and 5.64 nM, respectively. Sorafenib (IC_50_ > 2000 nM), tandutinib (IC50 > 10000 nM) and quizartinib (IC_50_ > 100 nM) (VANFLYTA^®^ in Japan) which have been reported as type II FLT3 inhibitors do not show potent inhibitory activity against FLT3-D835Y mutant, while quizartinib retains its activity with only reduced IC_50_ value against FLT3-D835Y compared with that against wild-type FLT3^10^^,^^28^. But most of our compounds were more potent against FLT3-TKD mutants than against wild-type FLT3, which was valuable to type II FLT3 kinase inhibitor development for AML therapeutics. Molecular docking analysis of **8r** into the active site of FLT3 kinase was performed, and the result revealed that compound **8r** tightly bound with the FLT3 active site. Based on docking study, newly introduced indazole and benzimidazole structures could properly interact with key amino acid residues as intended. Our research provided a theoretical rationale for the structural optimisation of benzimidazole derivatives as potent FLT3 inhibitors and demonstrated that compound **8r** could be a valuable scaffold for AML therapeutics due to its strong inhibitory activity against FLT3 and FLT3 mutants correlated with the secondary resistance mechanism of AML.

## 4. Materials and methods

### 4.1. General chemical methods

All chemicals were of reagent grade and were purchased from Aldrich (USA), Alfa aesar (USA), and TCI (JAPAN). Separation of the compounds by column chromatography was carried out with silica gel 60 (200–300 mesh ASTM, E. Merck, Germany). The quantity of silica gel used was 50–200 times the weight charged on the column. Thin layer chromatography (TLC) was run on the silica gel-coated aluminium sheets (silica gel 60 GF254, E. Merck, Germany) and visualised under ultraviolet (UV) light (254 nm). ^1^H NMR and ^13 ^C NMR spectra were recorded on a Brucker model digital AVANCE III 400 MHz spectrometer at 25 °C using tetramethylsilane (TMS) as an internal standard. High-resolution MS (HR/MS) experiments were conducted with a Finnigan LTQ Orbitrap mass spectrometer (Thermo Fisher Scientific Inc, MA, USA) operated in positive-ion electrospray mode.

#### 4.1.1. General procedure for the synthesis of methyl 1-(tetrahydro-2H-pyran-2-yl)-1H-indazole-6-carboxylate (2)

A mixture of methyl 1*H*-indazole-6-carboxylate **1** (1216 mg, 6.90 mmol), DHP (8.28 mmol) and PPTS (0.07 mmol) was reacted under microwave irradiation at 50 °C for 5 h to give methyl 1-(tetrahydro-2*H*-pyran-2-yl)-1*H*-indazole-6-carboxylate (**2**). After completion of reaction, the reaction mixture diluted with ethyl acetate and washed with saturated aqueous sodium bicarbonate. The organic layer dried over Na_2_SO_4_. The concentrated crude product was purified by flash column chromatography to afford Desired product as a yellow oil (1592.8 mg. 88.7%). ^1^H NMR (400 MHz, DMSO-*d*6) δ 8.63 (d, *J* = 0.9 Hz, 1H), 8.32 (dd, *J* = 2.3, 1.0 Hz, 1H), 7.84 (dd, *J* = 8.8, 0.9 Hz, 1H), 7.58 (dd, *J* = 8.8, 1.4 Hz, 1H), 5.81 (dd, *J* = 9.5, 2.8 Hz, 1H), 4.00 (dtd, *J* = 8.8, 3.7, 1.6 Hz, 1H), 3.88 (s, 3H), 3.74 (ddd, *J* = 11.5, 8.2, 6.2 Hz, 1H), 2.24–2.14 (m, 1H), 2.09 (dt, *J* = 9.0, 3.4 Hz, 1H), 2.00–1.93 (m, 1H), 1.80–1.70 (m, 1H), 1.65–1.57 (m, 2H). LC/MS (ESI^+^, m/z) calcd for C_13_H_16_N_2_O_3_Na [M + Na]^+^: 283.1053, found 283.3543.

#### 4.1.2. General procedure for the synthesis of (1-(tetrahydro-2H-pyran-2-yl)-1H-indazol-6-yl)methanol (3)

To a solution of methyl 1-(tetrahydro-2*H*-pyran-2-yl)-1*H*-indazole-6-carboxylate (1592.8 mg, 6.12 mmol) in anhydrous THF (30.6 mL) was added 2.0 M LAH in THF (3.4 mL) dropwise. After completion of reaction, a solution of 1 N aqueous sodium hydroxide solution was added to the residue and was extracted with ethyl acetate. The organic layer was washed with saturated saline solution, and dried over anhydrous sodium sodium sulphate. The solvent was distilled off under reduced pressure. The title crude compound (1335.1 mg, 93.9%) was obtained as a solid. ^1^H NMR (400 MHz, DMSO-*d*6) δ 8.05 (d, *J* = 0.5 Hz, 1H), 7.72–7.67 (m, 1H), 7.65 (d, *J* = 0.8 Hz, 1H), 7.16–7.10 (m, 1H), 5.82 (dd, *J* = 9.7, 2.5 Hz, 1H), 5.34 (t, *J* = 5.7 Hz, 1H), 4.65 (d, *J* J = 5.5 Hz, 2H), 3.91–3.85 (m, 1H), 3.73 (ddd, *J* = 11.4, 8.0, 5.9 Hz, 1H), 2.47–2.38 (m, 1H), 2.03 (ddt, *J* = 10.1, 7.9, 4.3 Hz, 1H), 1.99–1.93 (m, 1H), 1.81–1.70 (m, 1H), 1.62–1.54 (m, 2H). LC/MS (ESI^+^, m/z) calcd for C_13_H_16_N_2_O_2_Na [M + Na]^+^: 255.1104, found 255.0434.

#### 4.1.3. General procedure for the synthesis of 1-(tetrahydro-2H-pyran-2-yl)-1H-indazole-6-carbaldehyde (4)

To the stirred solution of (1-(tetrahydro-2*H*-pyran-2-yl)-1*H*-indazol-6-yl)methanol (1335.1 mg, 5.75 mmol) in DCM (8.3 mL) and THF (8.3 mL) was added Dess Martin Periodinane (1.437.9 mg, 5.75 mmol) and stirred at room temperature for 16 h. DCM was added and filtered through celite. Filtrate was evaporated under vacuum to afford the title compound (1.4 g). ^1^H NMR (400 MHz, DMSO-*d*6) δ 10.18–10.15 (m, 1H), 8.41 (d, *J* = 1.0 Hz, 1H), 8.29 (d, *J* = 0.6 Hz, 1H), 7.98 (d, *J* = 8.3 Hz, 1H), 7.70 (dd, *J* = 8.3, 1.2 Hz, 1H), 6.03 (dd, *J* = 9.6, 2.4 Hz, 1H), 3.93 (ddd, *J* = 7.5, 3.5, 1.8 Hz, 1H), 3.81 (ddd, *J* = 11.5, 7.9, 5.7 Hz, 1H), 2.48 − 2.40 (m, 1H), 2.10–2.01 (m, 2H), 1.84–1.74 (m, 1H), 1.63 (tt, *J* = 8.3, 3.9 Hz, 2H). LC/MS (ESI^+^, m/z) calcd for C_13_H_14_N_2_O_2_Na [M + Na]^+^: 253.0947, found 253.1530.

#### 4.1.4. General procedure for the synthesis of 6–(5-nitro-1H-benzo[d]imidazol-2-yl)-1-(tetrahydro-2H-pyran-2-yl)-1H-indazole (5)

A mixture of 4-nitrobenzene-1,2-diamine (948.6 mg, 6.19 mmol), 1-(tetrahydro-2*H*-pyran-2-yl)-1*H*-indazole-6-carbaldehyde **4** (1426.3 mg, 6.19 mmol), NH_4_Cl (331.4 mg, 6.19 mmol) and EtOH (61.9 mg) was reacted at 80 °C for 2 h. After starting material disappear, the reaction mixture diluted with ethyl acetate and washed with saturated aqueous sodium bicarbonate. The organic layer dried over Na_2_SO_4_. The concentrated crude product was purified by flash column chromatography to afford desired product as a yellow solid (548.0 mg. 24.4%). m.p. 142–144 °C. ^1^H NMR (400 MHz, DMSO-*d*6) δ 13.74 (s, 1H), 8.59 (s, 1H), 8.52 (s, 1H), 8.24 (d, *J* = 0.6 Hz, 1H), 8.17 (dd, *J* = 8.9, 2.2 Hz, 1H), 8.05 (dd, *J* = 8.4, 1.3 Hz, 1H), 8.00 (dd, *J* = 8.5, 0.7 Hz, 1H), 7.82 (d, *J* = 8.7 Hz, 1H), 5.99 (dd, *J* = 9.5, 2.2 Hz, 1H), 3.95 (d, *J* = 12.1 Hz, 1H), 3.17 (d, *J* = 5.3 Hz, 2H), 2.14–2.01 (m, 2H), 1.87–1.75 (m, 1H), 1.65 (d, *J* = 3.4 Hz, 2H). ^13 ^C NMR (101 MHz, DMSO) δ 154.6, 147.4, 143.3, 139.8, 137.5, 135.7, 134.3, 129.5, 127.5, 125.9, 122.3, 120.5, 114.9, 109.8, 84.8, 67.2, 29.4, 25.3, 22.7. LC/MS (ESI^+^, m/z) calcd for C_19_H_17_N_5_O_3_ [M + H]^+^: 364.1410, found 364.2837.

#### 4.1.5. General procedure for the synthesis of 2–(1-(tetrahydro-2H-pyran-2-yl)-1H-indazol-6-yl)-1H-benzo[d]imidazol-5-amine (6)

A suspension of compound **5** (200 mg, 0.551 mmol) and 20 mg of Pd/C (10%) in 5.51 mL of EtOH was stirred for 16 h under H_2_. After filtering through celite, the solution was concentrated under reduced pressure to give compound **6** (121.2 mg, 66%). The title crude compound was used as a starting material for next step without further purification. m.p. 142–143 °C. ^1^H NMR (400 MHz, DMSO-*d*6) δ 12.47 (s, 1H), 8.40 (s, 1H), 8.16 (s, 1H), 7.95 (dd, *J* = 8.5, 1.0 Hz, 1H), 7.88 (d, *J* = 8.4 Hz, 1H), 7.33 (d, *J* = 6.8 Hz, 1H), 6.73 (s, 1H), 6.57 (d, *J* = 7.9 Hz, 1H), 5.93 (dd, *J* = 9.6, 2.1 Hz, 1H), 5.02 (s, 2H), 3.97 (d, *J* = 11.5 Hz, 1H), 3.88–3.78 (m, 1H), 2.05 (ddd, *J* = 12.5, 9.2, 6.0 Hz, 2H), 1.88–1.74 (m, 1H), 1.65 (dd, *J* = 8.0, 4.2 Hz, 2H), 1.27 (q, *J* = 7.1 Hz, 1H). ^13 ^C NMR (101 MHz, DMSO) δ 153.2, 144.7, 141.8, 140.1, 134.1, 133.5, 131.2, 130.0, 129.4, 124.6, 121.7, 120.0, 112.7, 112.2, 84.7, 67.2, 29.4, 25.3, 22.8. LC/MS (ESI^+^, m/z) calcd for C_19_H_19_N_5_O [M + H]^+^: 334.1668, found 334.3547.

#### 4.1.6. General procedure for the synthesis of intermediates (7a-7z)

##### 4.1.6.1. 3–(4-Methylpiperazin-1-yl)-N-(2–(1-(tetrahydro-2H-pyran-2-yl)-1H-indazol-6-yl)-1H-benzo[d]imidazol-5-yl)-5-(trifluoromethyl)benzamide (7a)

To a solution of compound **6** (6.80 mg, 0.0204 mmol) in THF (0.204 mL) were added EDC (5.87 mg, 0.0306 mmol), HOBt (3.75 mg, 0.0245 mmol), DIPEA (0.0055 mL) and benzoic acid (7.05 mg, 0.0244 mmol). The reaction was stirred at rt for overnight. After completion of the reaction, the mixture was cooled to ambient temperature and solvent was removed *in vacuo*. The reaction mixture diluted with ethyl acetate and washed with saturated aqueous sodium bicarbonate. The organic layer dried over Na_2_SO_4_. The concentrated crude product was purified by flash column chromatography to afford desired product as a pure solid (4.6 mg). The title compound mixture was isolated as pure solid in 37.4% yield. LC/MS (ESI^+^, m/z) calcd for C_32_H_32_F_3_N_7_O_5_ [M + H]^+^: 604.2648, found 604.5891.

##### 4.1.6.2. 3–(4-Methyl-1H-imidazol-1-yl)-N-(2–(1-(tetrahydro-2H-pyran-2-yl)-1H-indazol-6-yl)-1H-benzo[d]imidazol-5-yl)-5-(trifluoromethyl)benzamide (7b)

The title compound mixture was isolated as pure solid in 8.6% yield by procedures similar to those described in **7a**. LC/MS (ESI^+^, m/z) calcd for C_32_H_26_F_3_N_7_O_2_ [M + H]^+^: 586.2178, found 586.4406.

##### 4.1.6.3. 3–(2-Methyl-1H-imidazol-1-yl)-N-(2–(1-(tetrahydro-2H-pyran-2-yl)-1H-indazol-6-yl)-1H-benzo[d]imidazol-5-yl)-5-(trifluoromethyl)benzamide (7c)

The title compound mixture was isolated as pure solid in 43.0% yield by procedures similar to those described in **7a**. LC/MS (ESI^+^, m/z) calcd for C_31_H_26_F_3_N_7_O_2_ [M + H]^+^: 586.2178, found 586.5126.

##### 4.1.6.4. 3-Morpholino-N-(2–(1-(tetrahydro-2H-pyran-2-yl)-1H-indazol-6-yl)-1H-benzo[d]imidazol-5-yl)-5-(trifluoromethyl)benzamide (7d)

The title compound mixture was isolated as pure solid in 36.8% yield by procedures similar to those described in **7a**. ^1^H NMR (400 MHz, DMSO-*d*6) δ 10.57 (s, 1H), 8.66 (s, 1H), 8.55 (s, 1H), 8.33 (s, 1H), 7.98 (d, *J* = 8.8 Hz, 1H), 7.92 (dd, *J* = 8.8, 1.4 Hz, 1H), 7.79 (s, 1H), 7.73 (m, 2H), 7.66 (d, *J* = 8.6 Hz, 1H), 7.43 (s, 1H), 5.85 (dd, *J* = 9.5, 2.7 Hz, 1H), 4.05 (d, *J* = 11.2 Hz, 1H), 3.80 (m, 5H), 3.34 (d, *J* = 4.8 Hz, 4H), 2.28–2.19 (m, 1H), 2.17–2.10 (m, 1H), 2.01 (m, 1H), 1.79 (m, 1H), 1.67–1.63 (m, 2H). LC/MS (ESI^+^, m/z) calcd for C_31_H_29_F_3_N_6_O_3_ [M + H]^+^: 591.2331, found 591.5899.

##### 4.1.6.5. 4–(4-Methylpiperazin-1-yl)-N-(2–(1-(tetrahydro-2H-pyran-2-yl)-1H-indazol-6-yl)-1H-benzo[d]imidazol-5-yl)-3-(trifluoromethyl)benzamide (7e)

The title compound mixture was isolated as pure solid in 49.4% yield by procedures similar to those described in **7a**. ^1^H NMR (400 MHz, DMSO-*d*6) δ 13.10 (s, 1H), 10.45 (s, 1H), 8.52 (s, 1H), 8.30–8.20 (m, 4H), 8.04 (dd, *J* = 8.5, 1.2 Hz, 1H), 7.98–7.93 (m, 1H), 7.65 (d, *J* = 8.4 Hz, 2H), 7.54 (d, *J* = 8.6 Hz, 1H), 5.97 (dd, *J* = 9.6, 2.2 Hz, 1H), 3.97 (d, *J* = 11.3 Hz, 1H), 3.88–3.80 (m, 1H), 3.00 (t, *J* = 4.7 Hz, 4H), 2.46 (s, 4H), 2.26 (s, 3H), 2.14–2.03 (m, 2H), 2.02–1.94 (m, 1H), 1.88–1.78 (m, 1H), 1.66 (s, 2H)

##### 4.1.6.6. 4-Morpholino-N-(2–(1-(tetrahydro-2H-pyran-2-yl)-1H-indazol-6-yl)-1H-benzo[d]imidazol-5-yl)-3-(trifluoromethyl)benzamide (7f)

The title compound mixture was isolated as pure solid in 12.7% yield by procedures similar to those described in **7a**. ^1^H NMR (400 MHz, DMSO-*d*6) δ 13.04 (s, 1H), 10.46 (s, 1H), 8.53 (s, 1H), 8.25 (m, 4H), 8.04 (m, 1H), 7.95 (d, *J* = 8.4 Hz, 1H), 7.69–7.52 (m, 3H), 5.97 (d, *J* = 9.6 Hz, 1H), 3.98 (d, *J* = 11.1 Hz, 1H), 3.84 (dd, *J* = 15.3, 9.2 Hz, 1H), 3.76 (d, *J* = 4.3 Hz, 4H), 3.35 (s, 1H), 2.99 (s, 4H), 2.52 (s, 3H), 2.08 (t, *J* = 13.4 Hz, 2H), 1.81 (d, *J* = 9.0 Hz, 1H), 1.66 (s, 2H). LC/MS (ESI^+^, m/z) calcd for C_31_H_29_F_3_N_6_O_3_ [M + H]^+^: 591.2331, found 591.4819.

##### 4.1.6.7. 1-Phenyl-N-(2–(1-(tetrahydro-2H-pyran-2-yl)-1H-indazol-6-yl)-1H-benzo[d]imidazol-5-yl)-5-(trifluoromethyl)-1H-pyrazole-4-carboxamide (7g)

The title compound mixture was isolated as pure solid in 85.5% yield by procedures similar to those described in **7a**. ^1^H NMR (400 MHz, DMSO-*d*6) δ 12.94–12.89 (s, 1H), 10.62–10.53 (s, 1H), 8.58 (s, 1H), 8.43 (d, *J* = 13.8 Hz, 2H), 8.34 (s, 1H), 8.21–8.11 (s, 1H), 7.97–7.91 (m, 1H), 7.87 (d, *J* = 8.8 Hz, 1H), 7.68–7.50 (m, 1H), 7.40 (d, *J* = 8.6 Hz, 1H), 5.79 (m, 1H), 4.06–3.99 (m, 1H), 3.81–3.71 (m, 1H), 2.27–2.17 (m, 1H), 2.14–2.05 (m, 1H), 2.02–1.93 (m, 1H), 1.82–1.71 (m, 1H), 1.66–1.57 (m, 2H). LC/MS (ESI^+^, m/z) calcd for C_30_H_24_F_3_N_7_O_2_ [M + H]^+^: 572.2022, found 572.4773.

##### 4.1.6.8. 5-(tert-Butyl)-N-(2–(1-(tetrahydro-2H-pyran-2-yl)-1H-indazol-6-yl)-1H-benzo[d]imidazol-5-yl)isoxazole-3-carboxamide (7h)

The title compound mixture was isolated as pure solid in 32.1% yield by procedures similar to those described in **7a**. ^1^H NMR (400 MHz, DMSO-*d*6) δ 12.95 (s, 1H), 10.67 (s, 1H), 8.60 (s, 1H), 8.43 (s, 1H), 8.24–8.16 (s, 1H), 7.96–7.87 (m, 2H), 7.65 (d, 1H), 7.53 (dd, *J* = 8.7, 1.9 Hz, 1H), 6.73 (d, *J* = 2.2 Hz, 1H), 5.81 (dd, *J* = 9.6, 2.5 Hz, 2H), 4.04 (d, *J* = 11.3 Hz, 1H), 3.83–3.73 (m, 1H), 2.22 (m, 1H), 2.15–2.08 (m, 1H), 2.05–1.97 (m, 1H), 1.82–1.74 (m, 1H), 1.65 (m, 2H), 1.39 (s, 9H). LC/MS (ESI^+^, m/z) calcd for C_27_H_28_N_6_O_3_ [M + H]^+^: 485.2301, found 485.1799.

##### 4.1.6.9. 4-Chloro-N-(2–(1-(tetrahydro-2H-pyran-2-yl)-1H-indazol-6-yl)-1H-benzo[d]imidazol-5-yl)-3-(trifluoromethyl)benzamide (7i)

The title compound mixture was isolated as pure solid in 17.9% yield by procedures similar to those described in **7a**. ^1^H NMR (400 MHz, DMSO-*d*6) δ 12.93 (s, 1H), 10.57 (s, 1H), 8.58 (s, 1H), 8.42 (m, = 7.4 Hz, 2H), 8.31 (dd, *J* = 8.4, 1.8 Hz, 1H), 8.23–8.14 (s, sH), 7.94 (m, 2H), 7.87 (d, *J* = 8.7 Hz, 1H), 7.66–7.55 (dd, 1H), 7.55–7.47 (d, *J* = 8.8 Hz, 1H), 5.80 (dd, *J* = 9.6, 2.7 Hz, 1H), 4.03 (d, *J* = 11.2 Hz, 1H), 3.80–3.72 (m, 1H), 2.22 (m, 1H), 2.13–2.06 (m, 1H), 2.04–1.90 (m, 2H), 1.76 (m, 1H), 1.63 (m, 1H). LC/MS (ESI^+^, m/z) calcd for C_27_H_21_ClF_3_N_5_O_2_ [M + H]^+^: 540.1414, found 540.4564.

##### 4.1.6.10. 3,4-Dichloro-N-(2–(1-(tetrahydro-2H-pyran-2-yl)-1H-indazol-6-yl)-1H-benzo[d]imidazol-5-yl)benzamide (7j)

The title compound mixture was isolated as pure solid in 30.0% yield by procedures similar to those described in **7a**. LC/MS (ESI^+^, m/z) calcd for C_26_H_21_Cl_2_N_5_O_2_ [M + H]^+^: 506.1151, found 506.4985.

##### 4.1.6.11. (E)-3–(4-Methoxyphenyl)-N-(2–(1-(tetrahydro-2H-pyran-2-yl)-1H-indazol-6-yl)-1H-benzo[d]imidazol-5-yl)acrylamide (7k)

The title compound mixture was isolated as pure solid in 78.8% yield by procedures similar to those described in **7a**. **^1^H NMR** (400 MHz, DMSO-*d*6) δ 12.97 (s, 1H), 10.24–10.14 (s, 1H), 8.49 (s, 1H), 8.35–8.19 (s, 1H), 8.19 (s, 1H), 8.00 (d, *J* = 7.4 Hz, 1H), 7.92 (d, *J* = 8.4 Hz, 1H), 7.64–7.30 (m, 5H), 7.02 (d, *J* = 8.8 Hz, 2H), 6.75 (d, *J* = 15.7 Hz, 2H), 5.98–5.91 (m, 1H), 3.96 (d, *J* = 11.2 Hz, 1H), 3.84 (d, *J* = 7.0 Hz, 1H), 3.81 (s, 3H), 2.13–1.87 (m, 3H), 1.79 (m, 1H), 1.64 (s, 2H). LC/MS (ESI^+^, m/z) calcd for C_29_H_27_N_5_O_3_ [M + H]^+^: 494.2192, found 494.2188.

##### 4.1.6.12. General procedure for the synthesis of 1–(5-(tert-butyl)isoxazol-3-yl)-3–(2-(1-(tetrahydro-2H-pyran-2-yl)-1H-indazol-6-yl)-1H-benzo[d]imidazol-5-yl)urea (7l–m)

5-(tert-butyl)isoxazol-3-amine (10.1 mg, 0.072 mmol) was desolved in THF (0.36 mL), and DIPEA (15.3 µl, 0.072 mmol) was added. The reaction mixture was cooled to 0 °C, and 4-nitrophenyl carbonochloridate (8.71 mg, 0.0432 mmol) was added. The reaction mixture was stirred at 0 °C for 30 min, then **compound 5** (12 mg, 0.036 mmol) and DIPEA (15.3 µl, 0.072 mmol) were added. The reaction mixture was stirred at 50 °C for 16 h. After termination, the reaction mixture was cooled to ambient temperature, quenched with MeOH and concentrated. The concentrated crude product was purified by flash column chromatography to afford desired product as a pure solid (4.5 mg). The title compound mixture was isolated as pure solid in 25.0% yield. ^1^H NMR (400 MHz, DMSO-*d*6) δ 12.82 (s, 1H), 9.46 (s, 1H), 8.92–8.79 (s, 1H), 8.57–8.09 (s, 2H), 7.96–7.82 (m, 3H), 7.58–7.45 (d, *J* = 8.5 Hz, 1H), 7.23–7.04 (dd, *J* = 8.6, 1.9 Hz, 1H), 6.53 (s, 1H), 5.79 (m, 1H), 4.56 (t, *J* = 5.5 Hz, 1H), 4.44 (t, *J* = 5.5 Hz, 1H), 4.02 (d, *J* = 11.2 Hz, 1H), 3.79–3.72 (m, 1H), 2.26–2.15 (m, 1H), 2.13–2.06 (m, 1H), 1.97 (m, 2H), 1.31 (s, 9H). LC/MS (ESI^+^, m/z) calcd for C_27_H_29_N_7_O_3_ [M + H]^+^: 500.2410, found 500.4486.

##### 4.1.6.13. 1–(3,4-Dichlorophenyl)-3–(2-(1-(tetrahydro-2H-pyran-2-yl)-1H-indazol-6-yl)-1H-benzo[d]imidazol-5-yl)urea (7m)

The title compound mixture was isolated as pure solid in 41.6% yield by procedures similar to those described in **7l**. ^1^H NMR (400 MHz, DMSO-*d*6) δ 12.81 (s, 1H), 8.97 (s, 1H), 8.88–8.74 (s, 1H), 8.57 (s, 1H), 8.40 (s, 1H), 7.92–7.81 (m, 4H), 7.58–7.45 (d, *J* = 8.6 Hz, 1H), 7.53 (d, *J* = 8.8 Hz, 1H), 7.36 (dd, *J* = 8.8, 2.5 Hz, 1H), 7.27–7.06 (dd, *J* = 8.6, 1.6 Hz, 1H), 5.79 (dd, *J* = 9.6, 2.6 Hz, 1H), 4.02 (d, *J* = 12.1 Hz, 1H), 3.80–3.71 (m, 1H), 2.21 (m, 1H), 2.14–2.06 (m, 1H), 2.03–1.95 (m, 1H), 1.81–1.72 (m, 1H), 1.63 (m, 2H). LC/MS (ESI^+^, m/z) calcd for C_26_H_22_Cl_2_N_6_O_2_ [M + H]^+^: 521.1260, found 521.0828.

##### 4.1.6.14. 3–(3-(Dimethylamino)pyrrolidin-1-yl)-N-(2–(1-(tetrahydro-2H-pyran-2-yl)-1H-indazol-6-yl)-1H-benzo[d]imidazol-5-yl)-5-(trifluoromethyl)benzamide (7n)

The title compound mixture was isolated as pure solid in 52.3% yield by procedures similar to those described in **7a**. m.p. 138–168 °C. ^1^H NMR (400 MHz, DMSO-*d*6) δ 13.04 (s, 1H), 10.39 (s, 1H), 8.52 (s, 1H), 8.28 (s, 1H), 8.24–8.18 (s, 1H), 8.07–8.01 (m, 1H), 7.96 (d, *J* = 8.4 Hz, 1H), 7.69–7.58 (m, 1H), 7.58–7.48 (m, 2H), 7.36 (s, 1H), 6.96 (s, 1H), 6.02–5.94 (m, 1H), 3.98 (d, *J* = 11.4 Hz, 1H), 3.83 (m, 1H), 3.62–3.54 (m, 2H), 3.21–3.16 (m, 1H), 2.89–2.82 (m, 1H), 2.25 (s, 6H), 2.22 (m, 2H), 2.12–2.04 (m, 2H), 1.97–1.77 (m, 3H), 1.66 (s, 2H). LC/MS (ESI^+^, m/z) calcd for C_33_H_34_F_3_N_7_O_2_ [M + H]^+^: 618.2804, found 618.3084.

##### 4.1.6.15. 3–(3-(Diethylamino)pyrrolidin-1-yl)-N-(2–(1-(tetrahydro-2H-pyran-2-yl)-1H-indazol-6-yl)-1H-benzo[d]imidazol-5-yl)-5-(trifluoromethyl)benzamide (7o)

The title compound mixture was isolated as pure solid in 45.9% yield by procedures similar to those described in **7a**. ^1^H NMR (400 MHz, DMSO-*d*6) δ 12.91 (s, 1H), 10.34 (s, 1H), 8.58 (s, 1H), 8.43 (s, 1H), 8.21–8.13 (d, *J* = 1.8 Hz, 1H), 7.97–7.91 (m, 1H), 7.87 (d, *J* = 8.8 Hz, 1H), 7.65–7.52 (d, *J* = 8.7 Hz, 1H), 7.59–7.47 (m, 2H), 7.34 (s, 1H), 6.93 (s, 1H), 5.80 (dd, *J* = 9.6, 2.6 Hz, 1H), 4.03 (d, *J* = 12.1 Hz, 1H), 3.80–3.72 (m, 1H), 3.65–3.58 (m, 1H), 2.72–2.55 (m, 4H), 2.26–2.17 (m, 2H), 2.14–2.06 (m, 1H), 1.93 (m, 3H), 1.76 (m, 2H), 1.67–1.58 (m, 2H), 1.45 (m, 1H), 1.36–1.27 (m, 1H), 1.00 (t, *J* = 6.8 Hz, 6H). LC/MS (ESI^+^, m/z) calcd for C_35_H_38_F_3_N_7_O_2_ [M + H]^+^: 646.3117, found 646.0709.

##### 4.1.6.16. 3–(4-Cyclopropylpiperazin-1-yl)-N-(2–(1-(tetrahydro-2H-pyran-2-yl)-1H-indazol-6-yl)-1H-benzo[d]imidazol-5-yl)-5-(trifluoromethyl)benzamide (7p)

The title compound mixture was isolated as pure solid in 60.2% yield by procedures similar to those described in **7a**. ^1^H NMR (400 MHz, DMSO-*d*6) δ 13.04 (s, 1H), 10.46–10.40 (s, 1H), 8.52 (s, 1H), 8.28–8.18 (s, 2H), 8.03 (d, *J* = 7.9 Hz, 1H), 7.95 (d, *J* = 8.4 Hz, 1H), 7.77 (s, 1H), 7.70–7.50 (m, 3H), 7.39 (s, 1H), 5.97 (d, *J* = 9.6 Hz, 1H), 3.97 (d, *J* = 11.4 Hz, 1H), 3.91–3.80 (m, 1H), 3.35–3.27 (m, 4H), 2.78–2.65 (m, 4H), 2.07 (m, 2H), 1.90–1.77 (m, 1H), 1.74–1.61 (m, 3H), 1.20 (ddd, *J* = 17.7, 15.6, 11.0 Hz, 1H), 0.51–0.44 (m, 2H), 0.42–0.33 (m, 2H). LC/MS (ESI^+^, m/z) calcd for C_34_H_34_F_3_N_7_O_2_ [M + H]^+^: 630.2804, found 630.3760.

##### 4.1.6.17. 3-((4-Cyclopropylpiperazin-1-yl)methyl)-N-(2–(1-(tetrahydro-2H-pyran-2-yl)-1H-indazol-6-yl)-1H-benzo[d]imidazol-5-yl)-5-(trifluoromethyl)benzamide (7q)

The title compound mixture was isolated as pure solid in 20.0% yield by procedures similar to those described in **7a**. ^1^H NMR (400 MHz, DMSO-*d*6) δ 13.06–13.04 (s, 1H), 10.57–10.51 (s, 1H), 8.54–8.51 (s, 1H), 8.31–8.20 (m, 4H), 8.03 (dd, *J* = 11.5, 4.3 Hz, 1H), 7.96 (d, *J* = 8.4 Hz, 1H), 7.88 (s, 1H), 7.70–7.59 (d, *J* = 8.7 Hz, 1H), 7.59–7.51 (dd, *J* = 8.7, 1.8 Hz, 1H), 6.02–5.94 (m, 10H), 3.98 (d, *J* = 11.3 Hz, 1H), 3.89–3.81 (m, 1H), 3.67 (s, 2H), 2.58 (m, 4H), 2.41 (m, 5H), 2.13–2.03 (m, 2H), 1.82 (m, 1H), 1.74–1.52 (m, 4H), 0.46–0.37 (m, 2H), 0.29 (d, 2H). LC/MS (ESI^+^, m/z) calcd for C_35_H_36_F_3_N_7_O_2_ [M + H]^+^: 644.2961, found 644.4907.

##### 4.1.6.18. 3-((4-Ethylpiperazin-1-yl)methyl)-N-(2–(1-(tetrahydro-2H-pyran-2-yl)-1H-indazol-6-yl)-1H-benzo[d]imidazol-5-yl)-5-(trifluoromethyl)benzamide (7r)

The title compound mixture was isolated as pure solid in 29.5% yield by procedures similar to those described in **7a**. ^1^H NMR (400 MHz, DMSO-*d*6) δ 13.02 (s, 1H), 10.50 (s, 1H), 8.48 (s, 1H), 8.28–8.14 (m, 4H), 8.02–7.96 (m, 1H), 7.91 (d, *J* = 8.4 Hz, 1H), 7.84 (s, 1H), 7.66 (d, *J* = 8.7 Hz, 1H), 7.54–7.45 (m, 2H), 5.99–5.90 (m, 1H), 3.93 (d, *J* = 11.4 Hz, 1H), 3.80 (dd, *J* = 15.3, 9.4 Hz, 1H), 3.64 (s, 2H), 2.45 (m, 10H), 2.36–2.24 (m, 4H), 2.10–2.00 (m, 2H), 1.84–1.74 (m, 1H), 1.62 (s, 2H). LC/MS (ESI^+^, m/z) calcd for C_34_H_36_F_3_N_7_O_2_ [M + H]^+^: 632.2961, found 632.8558.

##### 4.1.6.19. 3-((1-Methylpiperidin-4-yl)amino)-N-(2–(1-(tetrahydro-2H-pyran-2-yl)-1H-indazol-6-yl)-1H-benzo[d]imidazol-5-yl)-5-(trifluoromethyl)benzamide (7s)

The title compound mixture was isolated as pure solid in 41.0% yield by procedures similar to those described in **7a**. ^1^H NMR (400 MHz, DMSO-*d*6) δ 12.97 (s, 1H), 10.36 (s, 1H), 8.59 (d, *J* = 0.6 Hz, 1H), 8.45 (s, 1H), 8.20 (s, 1H), 7.96 (dd, *J* = 8.8, 1.2 Hz, 1H), 7.88 (dd, *J* = 8.8, 0.6 Hz, 1H), 7.61 (s, 1H), 7.52 (d, *J* = 7.8 Hz, 1H), 7.39 (d, *J* = 5.4 Hz, 2H), 7.05 (s, 1H), 6.31 (d, *J* = 8.0 Hz, 1H), 5.82 (dd, *J* = 9.6, 2.6 Hz, 1H), 4.10–4.01 (m, 1H), 3.83–3.73 (m, 1H), 3.37 (s, 2H), 2.75 (d, *J* = 11.6 Hz, 2H), 2.27 (dd, *J* = 12.6, 3.4 Hz, 1H), 2.20 (s, 3H), 2.14–1.98 (m, 4H), 1.93 (d, *J* = 10.8 Hz, 2H), 1.83–1.75 (m, 1H), 1.65 (dt, *J* = 11.7, 4.1 Hz, 2H), 1.46 (dd, *J* = 20.3, 10.1 Hz, 2H). LC/MS (ESI^+^, m/z) calcd for C_33_H_34_F_3_N_7_O_2_ [M + H]^+^: 618.2804, found 618.5965.

##### 4.1.6.20. 3-((1-Ethylpiperidin-4-yl)amino)-N-(2–(1-(tetrahydro-2H-pyran-2-yl)-1H-indazol-6-yl)-1H-benzo[d]imidazol-5-yl)-5-(trifluoromethyl)benzamide (7t)

The title compound mixture was isolated as pure solid in 25.8% yield by procedures similar to those described in **7a**. ^1^H NMR (400 MHz, DMSO-*d*6) δ 13.22 (s, 1H), 10.33 (s, 1H), 8.51 (s, 1H), 8.18 (s, 2H), 8.03 (dd, *J* = 8.5, 1.1 Hz, 1H), 7.91 (d, *J* = 8.4 Hz, 1H), 7.60 (d, *J* = 8.6 Hz, 1H), 7.53–7.47 (m, 1H), 7.37 (s, 2H), 7.02 (s, 1H), 6.29 (d, *J* = 8.0 Hz, 1H), 5.92 (dd, *J* = 9.6, 2.0 Hz, 1H), 3.94 (d, *J* = 11.6 Hz, 1H), 3.84–3.76 (m, 1H), 3.51–3.37 (m, 2H), 2.82 (d, *J* = 11.3 Hz, 2H), 2.33 (q, *J* = 7.2 Hz, 2H), 2.04 (dd, *J* = 20.7, 11.0 Hz, 4H), 1.94 (t, *J* = 12.7 Hz, 3H), 1.76 (dd, *J* = 23.7, 14.3 Hz, 2H), 1.63 (s, 2H), 1.00 (t, *J* = 7.2 Hz, 3H).

##### 4.1.6.21. 4-((1-Methylpiperidin-4-yl)oxy)-N-(2–(1-(tetrahydro-2H-pyran-2-yl)-1H-indazol-6-yl)-1H-benzo[d]imidazol-5-yl)-3-(trifluoromethyl)benzamide (7u)

The title compound mixture was isolated as pure solid in 41.3% yield by procedures similar to those described in **7a**. ^1^H NMR (400 MHz, DMSO-*d*6) δ 13.08 (s, 1H), 10.38 (s, 1H), 8.52 (s, 1H), 8.34–8.19 (m, 4H), 8.04 (dd, *J* = 8.5, 1.2 Hz, 1H), 7.95 (d, *J* = 8.4 Hz, 1H), 7.64 (d, *J* = 8.6 Hz, 1H), 7.54 (d, *J* = 8.6 Hz, 1H), 7.49 (d, *J* = 9.6 Hz, 1H), 5.97 (dd, *J* = 9.6, 2.1 Hz, 1H), 4.80 (s, 1H), 3.97 (d, *J* = 11.3 Hz, 1H), 3.90–3.80 (m, 1H), 2.51–2.45 (m, 2H), 2.33 (dd, *J* = 12.8, 5.2 Hz, 2H), 2.19 (d, *J* = 7.7 Hz, 3H), 2.07 (dd, *J* = 24.7, 11.1 Hz, 2H), 1.98 (dd, *J* = 16.4, 3.6 Hz, 3H), 1.83–1.72 (m, 3H), 1.66 (s, 2H).

##### 4.1.6.22. 3-((1-Methylpiperidin-3-yl)amino)-N-(2–(1-(tetrahydro-2H-pyran-2-yl)-1H-indazol-6-yl)-1H-benzo[d]imidazol-5-yl)-5-(trifluoromethyl)benzamide (7v)

The title compound mixture was isolated as pure solid in 43.8% yield by procedures similar to those described in **7a**. ^1^H NMR (400 MHz, DMSO-*d*6) δ 13.01 (s, 1H), 10.34 (s, 1H), 8.50 (s, 1H), 8.21 (m, 2H), 8.01 (dd, *J* = 11.3, 4.0 Hz, 1H), 7.93 (d, *J* = 8.4 Hz, 1H), 7.66 (d, *J* = 8.7 Hz, 1H), 7.56 (m, 1H), 7.48 (dd, *J* = 8.8, 1.9 Hz, 1H), 7.39 (s, 2H), 7.07 (s, 1H), 6.25 (dd, *J* = 8.3, 4.4 Hz, 1H), 5.99–5.92 (m, 1H), 3.96 (d, *J* = 11.1 Hz, 1H), 3.89–3.78 (m, 1H), 3.58 (dd, *J* = 8.7, 4.2 Hz, 1H), 2.81 (d, *J* = 9.6 Hz, 1H), 2.54 (dd, *J* = 15.0, 6.4 Hz, 1H), 2.12–1.92 (m, 4H), 1.89–1.75 (m, 3H), 1.62 (m, 4H), 1.29–1.17 (m, 2H). LC/MS (ESI^+^, m/z) calcd for C_33_H_34_F_3_N_7_O_2_ [M + H]^+^: 618.2804, found 618.3084.

##### 4.1.6.23. 4-((4-Ethylpiperazin-1-yl)methyl)-N-(2–(1-(tetrahydro-2H-pyran-2-yl)-1H-indazol-6-yl)-1H-benzo[d]imidazol-5-yl)-3-(trifluoromethyl)benzamide (7w)

The title compound mixture was isolated as pure solid in 48.5% yield by procedures similar to those described in **7a**. ^1^H NMR (400 MHz, DMSO-*d*6) δ 13.08 (s, 1H), 10.56–10.49 (s, 1H), 8.55–8.52 (s, 1H), 8.29–8.20 (m, 4H), 8.03 (m, 1H), 7.96 (m, 2H), 7.69–7.61 (d, *J* = 1.6 Hz, 1H), 7.58–7.52 (dd, *J* = 8.7, 1.9 Hz, 1H), 6.01–5.95 (m, 1H), 3.98–3.80 (m, 2H), 3.72 (s, 2H), 2.47 (m, 8H), 2.37 (m, 3H), 2.08 (m, 2H), 1.89–1.66 (m, 3H), 1.02 (t, *J* = 7.2 Hz, 3H). LC/MS (ESI^+^, m/z) calcd for C_34_H_36_F_3_N_7_O_2_ [M + H]^+^: 632.2961, found 632.6398.

##### 4.1.6.24. 3-((1-Methylpiperidin-4-yl)oxy)-N-(2–(1-(tetrahydro-2H-pyran-2-yl)-1H-indazol-6-yl)-1H-benzo[d]imidazol-5-yl)-5-(trifluoromethyl)benzamide (7x)

The title compound mixture was isolated as pure solid in 20.7% yield by procedures similar to those described in **7a**. LC/MS (ESI^+^, m/z) calcd for C_33_H_33_F_3_N_6_O_3_ [M + H]^+^: 619.2644, found 619.4607.

##### 4.1.6.25. 3-((4-Ethyl-3,3-dimethylpiperazin-1-yl)methyl)-N-(2–(1-(tetrahydro-2H-pyran-2-yl)-1H-indazol-6-yl)-1H-benzo[d]imidazol-5-yl)-5-(trifluoromethyl)benzamide (7y)

The title compound mixture was isolated as pure solid in 45.1% yield by procedures similar to those described in **7a**. ^1^H NMR (400 MHz, DMSO-*d*6) δ 13.17 (d, *J* = 3.8 Hz, 1H), 10.57 (d, *J* = 28.0 Hz, 1H), 8.57 (d, *J* = 8.0 Hz, 1H), 8.32 − 8.19 (m, 4H), 8.08–8.03 (m, 1H), 7.98–7.89 (m, 2H), 7.77–7.52 (m, 3H), 5.98 (d, *J* = 9.7 Hz, 1H), 3.97 (d, *J* = 11.3 Hz, 1H), 3.86 (dd, *J* = 9.3, 4.3 Hz, 1H), 3.65 (s, 2H), 2.35 (m, 2H), 2.20 (m, 1H), 2.07 (m, 3H), 1.82 (m, 1H), 1.67 (m, 2H), 1.15–0.92 (m, 12H), 0.88–0.81 (m, 2H). LC/MS (ESI^+^, m/z) calcd for C_36_H_40_F_3_N_7_O_2_ [M + H]^+^: 660.3274, found 660.5496.

##### 4.1.6.26. 3–(4-Ethyl-3,3-dimethylpiperazin-1-yl)-N-(2–(1-(tetrahydro-2H-pyran-2-yl)-1H-indazol-6-yl)-1H-benzo[d]imidazol-5-yl)-5-(trifluoromethyl)benzamide (7z)

The title compound mixture was isolated as pure solid in 71.3% yield by procedures similar to those described in **7a**. ^1^H NMR (400 MHz, DMSO-*d*6) δ 13.08 (d, *J* = 6.7 Hz, 1H), 10.45 (d, *J* = 26.0 Hz, 1H), 8.54 (d, *J* = 11.4 Hz, 1H), 8.29 (s, 1H), 8.21 (d, *J* = 5.0 Hz, 1H), 8.04 (d, *J* = 7.5 Hz, 1H), 7.95 (d, *J* = 8.4 Hz, 1H), 7.74 (s, 1H), 7.72–7.56 (m, 2H), 7.52 (d, *J* = 8.6 Hz, 1H), 7.39 (s, 1H), 5.97 (d, *J* = 9.6 Hz, 1H), 3.97 (d, *J* = 11.1 Hz, 1H), 3.84 (dd, *J* = 15.3, 9.1 Hz, 1H), 3.11 (s, 2H), 2.67 (s, 2H), 2.50 (d, *J* = 19.2 Hz, 3H), 2.41 (s, 2H), 2.08 (t, *J* = 13.1 Hz, 2H), 1.81 (d, *J* = 8.8 Hz, 1H), 1.65 (s, 2H), 1.09 (s, 6H), 1.03 (t, *J* = 6.8 Hz, 3H). LC/MS (ESI^+^, m/z) calcd for C_35_H_38_F_3_N_7_O_2_ [M + H]^+^: 646.3117, found 646.5791.

#### 4.1.7. General procedure for the synthesis of final product (8a–8z)

##### 4.1.7.1. N-(2-(1H-Indazol-6-yl)-1H-benzo[d]imidazol-5-yl)-3–(4-methylpiperazin-1-yl)-5-(trifluoromethyl)benzamide (8a)

To a solution of compound **7a** (4.6 mg 0.00762 mmol) was added 5% HCl in EtOH (0.0762 mL ∼ 0.174 mL). The reaction mixture was stirred at room temperature until Compound **7a** disappeared in TLC. After completion of the reaction, solvent was removed *in vacuo*. The reaction mixture diluted with ethyl acetate and washed with 1 M NaOH aqueous solution. The organic layer dried over Na_2_SO_4_. The concentrated crude product was purified by flash column chromatography to afford desired product as a pure solid (2.6 mg). The title compound mixture was isolated as pure solid in 50.0% yield. m.p. 228–230 °C. ^1^H NMR (400 MHz, DMSO-*d*6) δ 13.42 (s, 1H), 13.05 (s, 1H), 10.42 (s, 1H), 8.36 (s, 1H), 8.26–8.13 (m, 2H), 7.98 (s, 1H), 7.93 (s, 1H), 7.78 (s, 1H), 7.67 (s, 1H), 7.65–7.47 (m, 2H), 7.40 (s, 1H), 3.36 (m, 4H), 2.54 (m, 4H), 2.27 (s, 3H). ^13 ^C NMR (101 MHz, DMSO-*d*6) δ 164.9, 152.3, 151.8, 144.3, 141.2, 141.2, 140.5, 137.5, 135.5, 134.7, 134.7, 134.2, 132.6, 126.0, 121.6, 121.6, 119.6, 119.0, 119.0, 117.8, 114.0, 108.3, 54.9, 47.9, 46.2. HRMS (ESI^+^) calcd for C_27_H_24_F_3_N_7_O [M + H]^+^: 520.2073, found 520.1105.

##### 4.1.7.2. N-(2-(1H-Indazol-6-yl)-1H-benzo[d]imidazol-5-yl)-3–(4-methyl-1H-imidazol-1-yl)-5-(trifluoromethyl)benzamide (8b)

The title compound mixture was isolated as pure solid in 8.6% yield by procedures similar to those described in **8a**. m.p. 239–241 °C. ^1^H NMR (400 MHz, DMSO-*d*6) δ 13.42 (s, 1H), 13.04 (s, 1H), 10.59 (d, *J* = 23.7 Hz, 1H), 8.52 (s, 1H), 8.45 (d, *J* = 1.4 Hz, 1H), 8.36 (d, *J* = 12.2 Hz, 1H), 8.25 (d, *J* = 17.4 Hz, 2H), 8.19 (d, *J* = 8.2 Hz, 1H), 7.98 (s, 1H), 7.94 (d, *J* = 8.4 Hz, 1H), 7.76 (s, 1H), 7.70 (d, *J* = 8.7 Hz, 1H), 7.59 (s, 1H), 7.51 (d, *J* = 7.4 Hz, 1H), 2.23 (d, *J* = 0.8 Hz, 3H). ^13 ^C NMR (101 MHz, DMSO-*d*6) δ 161.4, 152.9, 145.7, 142.3, 140.5, 139.4, 138.4, 138.2, 137.6, 136.8, 136.3, 136.0, 133.3, 131.6, 128.7, 127.4, 124.0, 121.6, 121.6, 121.6, 119.5, 118.8, 113.9, 111.7, 109.2, 14.5. HRMS (ESI^+^) calcd for C_26_H_18_F_3_N_7_O [M + H]^+^: 502.1603, found 502.0331.

##### 4.1.7.3. N-(2-(1H-Indazol-6-yl)-1H-benzo[d]imidazol-5-yl)-3–(2-methyl-1H-imidazol-1-yl)-5-(trifluoromethyl)benzamide (8c)

To a solution of compound **7c** (10.2 mg 0.01742 mmol) and CH_2_Cl_2_ (0.1742 mL), was added 20% TFA (0.0348 mL). The reaction mixture was stirred at room temperature until Compound **7c** disappeared in TLC. After completion of the reaction, solvent was removed *in vacuo*. The reaction mixture diluted with ethyl acetate and washed with saturated aqueous sodium bicarbonate. The organic layer dried over Na_2_SO_4_. The concentrated crude product was purified by flash column chromatography to afford desired product as a pure solid (2.1 mg). The title compound mixture was isolated as pure solid in 10.0% yield. m.p. 251–253 °C. ^1^H NMR (400 MHz, DMSO-*d*6) δ 13.41 (s, 1H), 13.05 (d, *J* = 13.6 Hz, 1H), 10.62 (s, 1H), 8.40 (d, *J* = 9.3 Hz, 2H), 8.34 (s, 1H), 8.27 (d, *J* = 1.7 Hz, 1H), 8.17 (d, *J* = 3.7 Hz, 1H), 7.99–7.96 (m, 1H), 7.94 (d, *J* = 8.4 Hz, 1H), 7.69 (d, *J* = 8.8 Hz, 1H), 7.62–7.57 (m, 1H), 7.55 (d, *J* = 1.4 Hz, 1H), 7.50 (dd, *J* = 8.6, 1.9 Hz, 1H), 7.02 (d, *J* = 1.4 Hz, 1H), 2.40 (s, 3H). ^13 ^C NMR (101 MHz, DMSO-*d*6) δ 165.4, 152.5, 147.4, 144.7, 141.0, 139.5, 138.6, 137.7, 135.3, 134.9, 134.6, 134.2, 132.3, 131.5, 130.7, 129.6, 121.8, 121.3, 120.2, 120.1, 119.3, 119.3, 114.7, 113.1, 108.4, 13.7. HRMS (ESI^+^) calcd for C_26_H_28_F_3_N_7_O [M + H]^+^: 502.1603, found 502.3932.

##### 4.1.7.4. N-(2-(1H-Indazol-6-yl)-1H-benzo[d]imidazol-5-yl)-3-morpholino-5-(trifluoromethyl)benzamide (8d)

The title compound mixture was isolated as pure solid in 99.0% yield by procedures similar to those described in **8a**. m.p. 195–196 °C. ^1^H NMR (400 MHz, DMSO-*d*6) δ 13.41 (s, 1H), 13.02 (s, 1H), 10.45–10.39 (s, 1H), 8.36 (s, 1H), 8.24–8.16 (s, 1H), 8.17 (s, 1H), 8.01–7.93 (m, 2H), 7.78 (s, 1H), 7.71–7.63 (m, 2H), 7.57–7.47 (s, 1H), 7.42 (s, 1H), 3.83–3.78 (m, 4H), 3.33 (s, 4H). ^13 ^C NMR (101 MHz, DMSO-*d*6) δ 164.8, 152.3, 152.0, 144.3, 141.2, 140.5, 137.5, 135.5, 134.6, 134.2, 128.4, 124.0, 121.6, 119.5, 119.0, 117.7, 116.5, 114.5, 114.4, 113.8, 111.7, 111.4, 108.3, 103.7, 66.4, 48.2. HRMS (ESI^+^) calcd for C_26_H_21_F_3_N_6_O_2_ [M + H]^+^: 507.1756, found 507.4708.

##### 4.1.7.5. N-(2-(1H-Indazol-6-yl)-1H-benzo[d]imidazol-5-yl)-4–(4-methylpiperazin-1-yl)-3-(trifluoromethyl)benzamide (8e)

The title compound mixture was isolated as pure solid in 76.1% yield by procedures similar to those described in **8c**. m.p. 160–161 °C. ^1^H NMR (400 MHz, DMSO-*d*6) δ 13.42 (s, 1H), 13.05–13.01 (s, 1H), 10.46–10.40 (s, 1H), 8.36 (d, *J* = 12.3 Hz, 1H), 8.31–8.23 (m, 3H), 8.17 (s, 1H), 7.98 (dd, *J* = 7.4 Hz, 1H), 7.93 (d, *J* = 8.5 Hz, 1H), 7.69–7.63 (m, 2H), 7.58–7.50 (d, *J* = 8.6 Hz, 1H), 3.01 (t, *J* = 4.6 Hz, 4H), 2.54 (t, 3H), 2.30 (s, 3H). ^13 ^C NMR (101 MHz, DMSO-*d*6) δ 164.2, 155.1, 141.1, 140.5, 135.6, 134.2, 133.3, 131.3, 128.4, 127.5, 125.8, 124.8, 124.5, 124.1, 123.1, 121.6, 119.5, 119.0, 116.3, 111.4, 108.3, 103.5, 55.2, 53.0, 46.0. HRMS (ESI^+^) calcd for C_27_H_24_F_3_N_7_O [M + H]^+^: 520.2073, found 520.4830.

##### 4.1.7.6. N-(2-(1H-Indazol-6-yl)-1H-benzo[d]imidazol-5-yl)-4-morpholino-3-(trifluoromethyl)benzamide (8f)

The title compound mixture was isolated as pure solid in 44.4% yield by procedures similar to those described in **8c**. m.p. 202–204 °C. ^1^H NMR (400 MHz, DMSO-*d*6) δ 13.39 (s, 1H), 12.98 (s, 1H), 10.44 (s, 1H), 8.32 (s, 1H), 8.28 (s, 1H), 8.23 (s, 1H), 8.15 (s, 1H), 7.96 (dd, *J* = 8.6 Hz, 1H), 7.91 (d, *J* = 8.4 Hz, 1H), 7.72–7.61 (m, 2H), 7.51 (m, 2H), 3.80–3.70 (t, 4H), 3.01–2.93 (t, 4H). ^13 ^C NMR (101 MHz, DMSO-*d*6) δ 164.2, 154.8, 152.5, 149.9, 147.6, 140.5, 134.2, 133.4, 131.6, 128.3, 127.4, 127.4, 125.8, 125.1, 124.8, 124.3, 124.0, 123.1, 121.6, 119.5, 116.8, 108.4, 66.9, 53.6. HRMS (ESI^+^) calcd for C_26_H_21_F_3_N_6_O_2_ [M + H]^+^: 507.1756, found 507.3987.

##### 4.1.7.7. N-(2-(1H-Indazol-6-yl)-1H-benzo[d]imidazol-5-yl)-1-phenyl-5-(trifluoromethyl)-1H-pyrazole-4-carboxamide (8g)

The title compound mixture was isolated as pure solid in 22.0% yield by procedures similar to those described in **8c**. m.p. 258–260 °C. ^1^H NMR (400 MHz, DMSO-*d*6) δ 13.39 (s, 1H), 13.01–12.96 (s, 1H), 10.61–10.52 (s, 1H), 8.35–8.31 (m, 2H), 8.21–8.10 (m, 2H), 7.95–7.91 (m, 2H), 7.66–7.39 (m, 7H). ^13 ^C NMR (101 MHz, DMSO-*d*6) δ 159.3, 152.3, 144.3, 141.1, 140.5, 140.3, 135.6, 134.6, 134.2, 133.8, 130.6, 129.9, 128.4, 126.5, 122.3, 121.6, 121.2, 119.5, 119.2, 116.9, 115.6, 108.3, 102.8. HRMS (ESI^+^) calcd for C_25_H_16_F_3_N_7_O [M + H]^+^: 488.1447, found 488.3489.

##### 4.1.7.8. N-(2-(1H-Indazol-6-yl)-1H-benzo[d]imidazol-5-yl)-5-(tert-butyl)isoxazole-3-carboxamide (8 h)

The title compound mixture was isolated as pure solid in 84.2% yield by procedures similar to those described in **8a**. m.p. 147–149 °C. ^1^H NMR (400 MHz, DMSO-*d*6) δ 13.40 (s, 1H), 13.01 (s, 1H), 10.70–10.61 (s, 1H), 8.33 (s, 1H), 8.21–8.15 (m, 2H), 7.96–7.91 (m, 2H), 7.63 (m, 1H), 7.52 (m, 1H), 6.71 (s, 1H), 1.36 (s, 9H). ^13 ^C NMR (101 MHz, DMSO) δ 159.7, 157.9, 153.0, 152.5, 141.4, 140.5, 135.5, 134.2, 128.3, 124.0, 121.6, 119.5, 119.5, 119.2, 116.2, 111.5, 103.5, 99.3, 33.1, 29.0. HRMS (ESI^+^) calcd for C_22_H_20_N_6_O_2_ [M + H]^+^: 401.1726, found 401.4507.

##### 4.1.7.9. N-(2-(1H-Indazol-6-yl)-1H-benzo[d]imidazol-5-yl)-4-chloro-3-(trifluoromethyl)benzamide (8i)

The title compound mixture was isolated as pure solid in 100.0% yield by procedures similar to those described in **8a**. m.p. 215–217 °C. ^1^H NMR (400 MHz, DMSO-*d*6) δ 13.41 (s, 1H), 13.04–13.01 (s, 1H), 10.62–10.56 (s, 1H) 8.45 (s, 1H), 8.37–8.32 (m, 2H), 8.26–8.25 (m, 2H), 8.00–7.92 (m, 3H), 7.69–7.48 (m, 2H). ^13 ^C NMR (101 MHz, DMSO-*d*6) δ 163.5, 152.4, 144.3, 141.3, 140.5, 135.5, 134.9, 134.5, 134.2, 133.9, 132.4, 128.4, 127.6, 124.0, 121.6, 119.5, 119.1, 117.6, 116.3, 111.5, 108.3, 103.6. HRMS (ESI^+^) calcd for C_22_H_13_ClF_3_N_5_O [M + H]^+^: 456.0839, found 456.2264.

##### 4.1.7.10. N-(2-(1H-Indazol-6-yl)-1H-benzo[d]imidazol-5-yl)-3,4-dichlorobenzamide (8j)

The title compound mixture was isolated as pure solid in 76.7% yield by procedures similar to those described in **8a**. m.p. 255–257 °C. ^1^H NMR (400 MHz, DMSO-*d*6) δ 13.39 (s, 1H), 13.01 (s, 1H), 10.47 (s, 1H), 8.33 (d, *J* = 13.5 Hz, 1H), 8.25– 8.15 (m, 3H), 7.97–7.91 (m, 3H), 7.84 (dd, *J* = 8.4, 1.6 Hz, 1H), 7.58 (dd, *J* = 8.6, 1.7 Hz, 1H), 7.48 (dd, *J* = 8.7, 2.0 Hz, 1H). ^13 ^C NMR (101 MHz, DMSO-*d*6) δ 163.5, 152.4, 141.2, 140.5, 136.0, 135.5, 134.7, 134.6, 134.2, 131.8, 131.2, 130.1, 128.5, 124.1, 124.0, 121.6, 119.5, 119.1, 117.6, 116.3, 108.3. HRMS (ESI^+^) calcd for C_21_H_13_Cl_2_N_5_O [M + H]^+^: 422.0575, found 422.3745.

##### 4.1.7.11. (E)-N-(2-(1H-Indazol-6-yl)-1H-benzo[d]imidazol-5-yl)-3–(4-methoxyphenyl)acrylamide (8k)

The title compound mixture was isolated as pure solid in 99.0% yield by procedures similar to those described in **8c**. m.p. 206–208 °C. ^1^H NMR (400 MHz, DMSO-*d*6) δ 13.39 (s, 1H), 12.97 (s, 1H), 10.19 (s, 1H), 8.32 (s, 1H), 8.18 (s, 1H), 7.95 (d, *J* = 7.7 Hz, 1H), 7.90 (d, *J* = 8.4 Hz, 1H), 7.59–7.48 (s, 5H), 7.29 (d, *J* = 7.8 Hz, 1H), 7.02 (d, *J* = 8.7 Hz, 2H), 6.75 (d, *J* = 15.6 Hz, 1H), 3.81 (s, 3H). ^13 ^C NMR (101 MHz, DMSO-*d*6) δ 164.2, 161.0, 152.1, 140.6, 140.5, 140.0, 137.5, 136.8, 136.1, 136.1, 134.3, 129.8, 127.9, 124.1, 124.0, 121.6, 120.5, 119.5, 119.5, 115.0, 108.4, 55.8. HRMS (ESI^+^) calcd for C_24_H_19_N_5_O_2_ [M + H]^+^: 410.1617, found 410.5261.

##### 4.1.7.12. 1–(2-(1H-Indazol-6-yl)-1H-benzo[d]imidazol-5-yl)-3–(5-(tert-butyl)isoxazol-3-yl)urea (8l)

The title compound mixture was isolated as pure solid in 72.2% yield by procedures similar to those described in **8c**. m.p. 184–186 °C. ^1^H NMR (400 MHz, DMSO-*d*6) δ 13.44 (s, 1H), 9.50 (s, 1H), 8.96 (s, 1H), 8.33 (s, 1H), 8.17 (s, 1H), 8.09 (s, 1H), 7.93 (s, 2H), 7.57 (d, *J* = 8.3 Hz, 1H), 7.15 (d, *J* = 8.5 Hz, 1H), 6.53 (s, 1H), 1.31 (s, 9H). ^13 ^C NMR (101 MHz, DMSO) δ 160.0, 152.6, 152.0, 149.0, 147.2, 146.9, 146.8, 140.6, 140.3, 136.6, 134.2, 130.0, 128.9, 123.5, 119.4, 113.5, 109.3, 92.9, 31.2, 28.9. HRMS (ESI^+^) calcd for C_22_H_21_N_7_O_2_ [M + H]^+^: 416.1835, found 416.4323.

##### 4.1.7.13. 1–(2-(1H-Indazol-6-yl)-1H-benzo[d]imidazol-5-yl)-3–(3,4-dichlorophenyl)urea (8m)

The title compound mixture was isolated as pure solid in 56.1% yield by procedures similar to those described in **8c**. m.p. 144–146 °C. ^1^H NMR (400 MHz, DMSO-*d*6) δ 13.36 (s, 1H), 12.88 (s, 1H), 9.08 (s, 1H), 9.00 (s, 1H), 8.31 (s, 1H), 8.14 (s, 1H), 7.97–7.84 (m, 4H), 7.57 (d, *J* = 8.6 Hz, 1H), 7.52 (dd, *J* = 8.8, 2.5 Hz, 1H), 7.36 (m, 1H), 7.07 (dd, *J* = 8.6, 1.9 Hz, 1H). ^13 ^C NMR (101 MHz, DMSO) δ 160.0, 140.5, 139.5, 138.8, 134.2, 131.7, 131.5, 131.1, 131.0, 129.7, 128.6, 128.6, 123.8, 121.5, 120.1, 119.7, 119.4, 119.3, 118.8, 114.9, 108.1. HRMS (ESI^+^) calcd for C_21_H_14_Cl_2_N_6_O [M + H]^+^: 437.0684, found 437.2479.

##### 4.1.7.14. N-(2-(1H-Indazol-6-yl)-1H-benzo[d]imidazol-5-yl)-3–(3-(dimethylamino)pyrrolidin-1-yl)-5-(trifluoromethyl)benzamide (8n)

The title compound mixture was isolated as pure solid in 11.0% yield by procedures similar to those described in **8c**. m.p. 283–285 °C. ^1^H NMR (400 MHz, DMSO-*d*6) δ 13.39 (s, 1H), 13.01 (s, 1H), 10.47 (s, 1H), 8.33 (d, *J* = 13.5 Hz, 1H), 8.25–8.15 (m, 3H), 7.97–7.91 (m, 3H), 7.84 (dd, *J* = 8.4, 1.6 Hz, 1H), 7.58 (dd, *J* = 8.6, 1.7 Hz, 1H), 7.48 (dd, *J* = 8.7, 2.0 Hz, 1H). ^13 ^C NMR (101 MHz, DMSO-*d*6) δ 165.2, 152.4, 148.1, 141.1, 140.5, 137.4, 135.6, 134.7, 134.2, 130.7, 128.3, 126.2, 124.0, 123.5, 121.5, 119.6, 119.0, 116.6, 114.4, 110.7, 110.0, 103.8, 65.5, 52.5, 47.4, 44.4, 30.1. HRMS (ESI^+^) calcd for C_28_H_26_F_3_N_7_O [M + H]^+^: 534.2229, found 534.4427.

##### 4.1.7.15. N-(2-(1H-Indazol-6-yl)-1H-benzo[d]imidazol-5-yl)-3–(3-(diethylamino)pyrrolidin-1-yl)-5-(trifluoromethyl)benzamide (8o)

The title compound mixture was isolated as pure solid in 24.6% yield by procedures similar to those described in **8c**. m.p. 221–223 °C. ^1^H NMR (400 MHz, DMSO-*d*6) δ 13.38 (s, 1H), 12.98 (s, 1H), 10.37–10.31 (s, 1H), 8.34–8.31 (s, 1H), 8.21–8.09 (m, 2H), 7.97 (dd, *J* = 11.4, 4.2 Hz, 1H), 7.91 (d, *J* = 8.1 Hz, 1H), 7.65–7.50 (d, *J* = 8.5 Hz, 1H), 7.56–7.46 (dd, *J* = 8.6, 1.6 Hz, 1H), 7.52–7.44 (m, 1H), 7.34 (s, 1H), 6.94 (s, 1H), 4.77 (s, 1H), 4.56 (m, 2H), 4.43 (t, *J* = 5.4 Hz, 4H), 2.67 (m, 4H), 1.09 (t, *J* = 7.0 Hz, 6H). ^13 ^C NMR (101 MHz, DMSO) δ 162.7, 158.9, 150.9, 147.5, 143.6, 141.2, 140.0, 140.0, 137.4, 136.3, 135.7, 135.6, 134.3, 130.0, 129.1, 126.2, 126.1, 119.1, 119.0, 114.4, 110.7, 60.3, 47.1, 43.8, 31.2, 14.4, 11.8. HRMS (ESI^+^) calcd for C_30_H_30_F_3_N_7_O [M + H]^+^: 562.2542, found 562.5664

##### 4.1.7.16. N-(2-(1H-Indazol-6-yl)-1H-benzo[d]imidazol-5-yl)-3–(4-cyclopropylpiperazin-1-yl)-5-(trifluoromethyl)benzamide (8p)

The title compound mixture was isolated as pure solid in 93.9% yield by procedures similar to those described in **8c**. m.p. 154–156 °C. ^1^H NMR (400 MHz, DMSO-*d*6) δ 13.41 (s, 1H), 13.01 (s, 1H), 10.43 (s, 1H), 8.36 (s, 1H), 8.19 (m, 2H), 7.99 (dd, *J* = 6.8, 1H), 7.93 (d, *J* = 8.5 Hz, 1H), 7.77 (s, 1H), 7.71–7.57 (m, 2H), 7.52 (d, *J* = 7.1 Hz, 1H), 7.39 (s, 1H), 3.33 (m, 5H), 2.76 (s, 4H), 0.46 (m, 4H). ^13 ^C NMR (101 MHz, DMSO-*d*6) δ 164.8, 151.9, 151.7, 140.5, 137.5, 134.2, 130.8, 130.4, 128.3, 126.0, 124.0, 124.0, 123.3, 121.6, 119.5, 117.9, 117.9, 117.9, 114.1, 114.1, 114.0, 108.4, 52.9, 47.9, 40.0, 38.5. **HRMS (ESI^+^)** calcd for C_29_H_26_F_3_N_7_O [M + H]^+^: 546.2229, found 546.5882.

##### 4.1.7.17. N-(2-(1H-Indazol-6-yl)-1H-benzo[d]imidazol-5-yl)-3-((4-cyclopropylpiperazin-1-yl)methyl)-5-(trifluoromethyl)benzamide (8q)

The title compound mixture was isolated as pure solid in 89.7% yield by procedures similar to those described in **8c**. m.p. 150–152 °C. ^1^H NMR (400 MHz, DMSO-*d*6) δ 13.41 (s, 1H), 13.04–13.01 (s, 1H), 10.56–10.50 (s, 1H), 8.37–8.34 (s, 1H), 8.26–8.17 (m, 4H), 7.98–7.93 (m, 2H), 7.88 (s, 1H), 7.68–7.50 (m, 2H), 3.67 (s, 2H), 2.58 (m, 4H), 2.41 (m, 5H), 0.41–0.29 (m, 4H). ^13 ^C NMR (101 MHz, DMSO-*d*6) δ 164.4, 158.4, 140.5, 136.7, 134.2, 132.6, 132.6, 132.6, 130.0, 129.8, 129.5, 129.5, 128.6, 128.3, 125.9, 125.4, 124.0, 123.2, 121.6, 119.5, 116.4, 108.4, 61.3, 53.1, 52.7, 38.4, 6.1. HRMS (ESI^+^) calcd for C_30_H_28_F_3_N_7_O [M + H]^+^: 560.2386, found 560.7390.

##### 4.1.7.18. N-(2-(1H-Indazol-6-yl)-1H-benzo[d]imidazol-5-yl)-3-((4-ethylpiperazin-1-yl)methyl)-5-(trifluoromethyl)benzamide (8r)

The title compound mixture was isolated as pure solid in 65.5% yield by procedures similar to those described in **8a**. m.p. 265–267 °C. ^1^H NMR (400 MHz, DMSO-*d*6) δ 10.46 (s, 1H), 8.35 (s, 1H), 8.27 (m, 2H), 8.15 (s, 1H), 8.13 (s, 1H), 7.99 (dd, *J* = 8.5, 1.3 Hz, 1H), 7.94 (d, *J* = 7.8 Hz, 1H), 7.87 (d, *J* = 8.5 Hz, 1H), 7.57 (d, *J* = 8.6 Hz, 1H), 7.46 (dd, *J* = 8.6, 1.6 Hz, 1H), 3.69 (s, 2H), 3.34 (s, 4H), 2.44 (s, 4H), 2.35–2.30 (m, 2H), 0.98 (m, 3H). ^13 ^C NMR (101 MHz, DMSO-*d*6) δ 164.4, 152.4, 141.1, 141.1, 136.6, 135.6, 134.6, 134.2, 132.5, 129.8, 129.5, 128.3, 125.9, 124.0, 123.4, 123.4, 123.4, 121.6, 119.5, 119.1, 116.4, 103.7, 61.4, 52.9, 52.7, 52.0, 12.3. HRMS (ESI^+^) calcd for C_29_H_28_F_3_N_7_O [M + H]^+^: 548.2386, found 548.4866.

##### 4.1.7.19. N-(2-(1H-Indazol-6-yl)-1H-benzo[d]imidazol-5-yl)-3-((1-methylpiperidin-4-yl)amino)-5-(trifluoromethyl)benzamide (8s)

The title compound mixture was isolated as pure solid in 96.7% yield by procedures similar to those described in **8a**. m.p. 175–177 °C. ^1^H NMR (400 MHz, DMSO-*d*6) δ 13.40 (s, 1H), 12.99 (s, 1H), 10.35 (s, 1H), 8.33 (s, 1H), 8.19–8.15 (m, 2H), 7.97–7.89 (m, 2H), 7.62–7.37 (m, 4H), 7.05 (s, 1H), 6.50–6.31 (d, 1H), 3.64 (m, 1H), 3.46–3.38 (m, 2H), 2.85 (d, 2H), 2.26 (s, 3H), 1.99–1.92 (m, 2H), 1.51–1.42 (m, 2H). ^13 ^C NMR (101 MHz, DMSO-*d*6) δ 165.3, 156.1, 154.8, 149.0, 141.1, 140.6, 137.7, 135.5, 134.3, 134.2, 128.4, 127.1, 126.2, 124.0, 121.6, 119.6, 119.5, 115.0, 111.2, 111.1, 110.7, 103.6, 60.2, 54.2, 46.1, 31.5. HRMS (ESI^+^) calcd for C_28_H_26_F_3_N_7_O [M + H]^+^: 534.2229, found 534.2986.

##### 4.1.7.20. N-(2-(1H-Indazol-6-yl)-1H-benzo[d]imidazol-5-yl)-3-((1-ethylpiperidin-4-yl)amino)-5-(trifluoromethyl)benzamide (8t)

The title compound mixture was isolated as pure solid in 21.2% yield by procedures similar to those described in **8c**. m.p. 166–168 °C. ^1^H NMR (400 MHz, DMSO-*d*6) δ 13.41 (s, 1H), 13.00 (s, 1H), 10.37–10.30 (s, 1H), 8.35 (s, 1H), 8.22–8.15 (s, 1H), 8.17 (s, 1H), 8.01–7.91 (m, 2H), 7.66–7.53 (d, *J* = 8.7 Hz, 1H), 7.57–7.47 (dd, *J* = 1.5 Hz, 1H), 7.40 (m, 2H), 7.06 (s, 1H), 6.34 (s, 1H), 4.58 (s, 1H), 2.51 (q, 2 h) 2.00–1.91 (m, 4H), 1.47 (s, 4H), 1.12 (t, *J* = 7.0 Hz, 3H). ^13 ^C NMR (101 MHz, DMSO-*d*6) δ 163.7, 150.8, 148.8, 139.1, 138.5, 134.8, 134.6, 134.2, 131.5, 129.0, 126.7, 125.7, 124.6, 121.6, 121.0, 120.3, 118.9, 117.6, 116.6, 116.4, 115.8, 113.6, 63.0, 53.1, 51.7, 30.4, 14.3. HRMS (ESI^+^) calcd for C_29_H_28_F_3_N_7_O [M + H]^+^: 548.2386, found 548.5326.

##### 4.1.7.21. N-(2-(1H-Indazol-6-yl)-1H-benzo[d]imidazol-5-yl)-3-((1-methylpiperidin-4-yl)oxy)-5-(trifluoromethyl)benzamide (8u)

The title compound mixture was isolated as pure solid in 85.9% yield by procedures similar to those described in **8a**. m.p. 293–295 °C. ^1^H NMR (400 MHz, DMSO-*d*6) δ 13.40 (s, 1H), 13.00 (s, 1H), 10.44 (s, 1H), 8.34 (s, 1H), 8.21 (s, 1H), 8.15 (s, 1H), 7.95 (dd, 1H), 7.91 (d, *J* = 8.4 Hz, 1H), 7.87 (s, 1H), 7.84 (s, 1H), 7.70–7.61 (m, 1H), 7.55 (dd, *J* = 10.4 Hz, 1H), 7.49 (s, 1H), 4.70–4.63 (m, 1H), 2.61 (d, *J* = 6.5 Hz, 2H), 2.28–2.17 (m, 5H), 1.95 (d, *J* = 13.4 Hz, 2H), 1.75–1.66 (m, 2H). ^13 ^C NMR (101 MHz, DMSO) δ 165.4, 158.1, 154.1, 148.1, 146.8, 144.9, 138.2, 134.6, 134.5, 134.2, 132.6, 132.6, 132.4, 132.0, 131.1, 130.6, 129.9, 125.2, 119.5, 115.4, 112.1, 110.9, 52.7, 46.3, 30.8, 29.5. HRMS (ESI^+^) calcd for C_28_H_25_F_3_N_6_O_2_ [M + H]^+^: 535.2069, found 535.3787.

##### 4.1.7.22. N-(2-(1H-Indazol-6-yl)-1H-benzo[d]imidazol-5-yl)-3-((1-methylpiperidin-3-yl)amino)-5-(trifluoromethyl)benzamide (8v)

The title compound mixture was isolated as pure solid in 100.0% yield by procedures similar to those described in **8a**. m.p. 213–214 °C. ^1^H NMR (400 MHz, DMSO-*d*6) δ 13.42 (s, 1H), 13.02 (s, 1H), 10.35 (s, 1H), 8.36 (s, 1H), 8.22 (s, 1H), 8.16 (s, 1H), 8.03–7.96 (m, 1H), 7.93 (d, *J* = 8.5 Hz, 1H), 7.66–7.59 (m, 1H), 7.57–7.48 (m, 1H), 7.41 (s, 2H), 7.09 (s, 1H), 6.29 (d, *J* = 8.3 Hz, 1H), 3.64–3.57 (m, 1H), 2.85 (d, *J* = 9.1 Hz, 1H), 2.59 (d, 1H), 2.22 (s, 3H), 2.02 (m, 1H), 1.91 (m, 2H), 1.74 (m, 1H), 1.66–1.57 (m, 1H), 1.28 (m, 1H). ^13 ^C NMR (101 MHz, DMSO-*d*6) δ 165.3, 152.3, 149.0, 141.1, 140.5, 137.7, 135.5, 134.8, 134.2, 130.3, 128.4, 126.1, 121.6, 119.5, 119.0, 116.4, 114.7, 111.4, 110.8, 110.8, 108.3, 103.6, 60.8, 55.7, 48.7, 46.5, 29.7, 23.7. HRMS (ESI^+^) calcd for C_28_H_26_F_3_N_7_O [M + H]^+^: 534.2229, found 534.3707.

##### 4.1.7.23. N-(2-(1H-Indazol-6-yl)-1H-benzo[d]imidazol-5-yl)-4-((4-ethylpiperazin-1-yl)methyl)-3-(trifluoromethyl)benzamide (8w)

The title compound mixture was isolated as pure solid in 66.8% yield by procedures similar to those described in **8c**. m.p. 164–166 °C. ^1^H NMR (400 MHz, DMSO-*d*6) δ 13.42 (s, 1H), 13.08–13.05 (s, 1H), 10.55–10.48 (s, 1H), 8.36–8.19 (m, 4H), 8.15 (s, 1H), 8.00–7.89 (m, 3H), 7.65–7.50 (m, 2H), 3.73 (s, 2H), 3.32 (m, 4H), 2.49 (m, 6H), 1.23 (s, 3H). ^13 ^C NMR (101 MHz, DMSO) δ 164.4, 156.8, 152.4, 141.2, 140.5, 134.8, 134.2, 132.1, 132.1, 131.2, 128.4, 127.9, 127.6, 125.7, 124.0, 123.3, 121.6, 119.5, 119.1, 116.4, 111.5, 103.6, 57.6, 52.0, 51.7, 29.5, 11.2. HRMS (ESI^+^) calcd for C_29_H_28_F_3_N_7_O [M + H]^+^: 548.2386, found 548.4866.

##### 4.1.7.24. N-(2-(1H-Indazol-6-yl)-1H-benzo[d]imidazol-5-yl)-4-((1-methylpiperidin-4-yl)oxy)-3-(trifluoromethyl)benzamide (8x)

The title compound mixture was isolated as pure solid in 60.4% yield by procedures similar to those described in **8c**. m.p. 293–295 °C. ^1^H NMR (400 MHz, DMSO-*d*6) δ 13.56 (s, 1H), 13.40–13.35 (s, 1H), 10.53–10.45 (s, 1H), 8.41 (d, *J* = 7.2 Hz, 1H), 8.36 (d, *J* = 8.5 Hz, 1H), 8.30 (d, *J* = 1.9 Hz, 1H), 8.21–8.15 (s, 1H), 8.12 (s, 1H), 8.00 (d, *J* = 6.1 Hz, 1H), 7.88 (d, *J* = 8.4 Hz, 1H), 7.61 (d, *J* = 8.6 Hz, 1H), 7.56 (dd, *J* = 10.7 Hz, 1H), 7.48 (d, *J* = 10.2 Hz, 1H), 4.17 (dd, *J* = 5.4 Hz, 1H), 3.15 (s, 3H), 2.27 (m, 2H), 2.00 (m, 2H), 1.79 (m, 2H), 1.44 (m, 2H). ^13 ^C NMR (101 MHz, DMSO-*d*6) δ 161.8, 153.5, 153.0, 142.1, 138.1, 137.3, 136.3, 135.8, 134.4, 134.1, 132.1, 131.5, 130.5, 128.3, 127.5, 125.4, 122.1, 117.8, 116.4, 114.9, 114.5, 111.3, 76.3, 53.8, 49.0, 29.5. HRMS (ESI^+^) calcd for C_28_H_25_F_3_N_6_O_2_ [M + H]^+^: 535.2069, found 535.4980.

##### 4.1.7.25. N-(2-(1H-Indazol-6-yl)-1H-benzo[d]imidazol-5-yl)-3-((4-ethyl-3,3-dimethylpiperazin-1-yl)methyl)-5-(trifluoromethyl)benzamide (8y)

The title compound mixture was isolated as pure solid in 100% yield by procedures similar to those described in **8c**. m.p. 255–257 °C. ^1^H NMR (400 MHz, DMSO-*d*6) δ 13.41 (s, 1H), 13.02 (s, 1H), 10.54–10.48 (s, 1H), 8.35 (s, 1H), 8.28–8.18 (m, 3H), 8.15 (s, 1H), 8.00–7.86 (m, 3H), 7.66 (d, *J* = 8.6 Hz, 1H), 7.50 (d, *J* = 8.3 Hz, 1H), 3.62 (s, 2H), 3.35–3.18 (m, 2H), 2.57 (m, 2H), 2.33 (m, 2H), 2.13 (m, 2H), 1.20 (s, 3H), 0.98 (m, 9H). ^13 ^C NMR (101 MHz, DMSO-*d*6) δ 164.4, 152.4, 141.5, 141.4, 141.2, 137.5, 136.7, 135.6, 134.6, 134.2, 133.8, 132.1, 129.8, 129.5, 128.4, 125.9, 123.2, 121.5, 119.5, 119.1, 116.3, 103.6, 61.1, 54.3, 54.1, 46.3, 43.0, 42.9, 14.6, 14.4. HRMS (ESI^+^) calcd for C_31_H_32_F_3_N_7_O [M + H]^+^: 576.2699, found 576.5820.

##### 4.1.7.26. N-(2-(1H-Indazol-6-yl)-1H-benzo[d]imidazol-5-yl)-3–(4-ethyl-3,3-dimethylpiperazin-1-yl)-5-(trifluoromethyl)benzamide (8z)

The title compound mixture was isolated as pure solid in 100% yield by procedures similar to those described in **8c**. m.p. 153–154 °C. ^1^H NMR (400 MHz, DMSO-*d*6) δ 13.41 (s, 1H), 13.03 (s, 1H), 10.43 (s, 1H), 8.35 (s, 1H), 8.27–8.12 (m, 2H), 7.98 (d, *J* = 8.2 Hz, 1H), 7.92 (d, *J* = 8.4 Hz, 1H), 7.75 (s, 1H), 7.66 (s, 1H), 7.52 (m, 2H), 7.40 (s, 1H), 3.35–3.23 (m, 2H), 3.16 (m, 2H), 2.76 (m, 2H), 1.24–1.02 (m, 11H). ^13 ^C NMR (101 MHz, DMSO-*d*6) δ 164.9, 151.6, 141.2, 139.1, 138.1, 137.6, 135.6, 134.6, 134.2, 130.8, 130.5, 128.4, 126.0, 124.0, 123.3, 121.5, 120.6, 119.5, 119.0, 117.9, 114.0, 111.3, 45.6, 34.7, 31.4, 29.0, 25.3, 22.5, 14.4. HRMS (ESI^+^) calcd for C_30_H_30_F_3_N_7_O [M + H]^+^: 562.6212, found 562.6833.

### 4.2. Evaluation of IC_50_ and selected kinase profiling

We used Reaction Biology Corp. *Kinase HotSpot^SM^* service (www.reactionbiology.com) for IC_50_ determination of all compounds and kinase profile. Assay protocol: In a final reaction volume of 25 μL, Peptide substrate, [EAIYAAPFAKKK], 5 µM, ATP 10 µM, FLT3(h) (5–10 mU) is incubated with 25 mM Tris pH 7.5, 0.02 mM EGTA, 0.66 mg/mL myelin basic protein, 10 mM Mg Acetate and [γ-33P-ATP] (specific activity approx. 500 cpm/pmol, concentration as required). The reaction is initiated by the addition of the Mg-ATP mix. After incubation for 40 min at room temperature, the reaction is stopped by the addition of 5 μL of a 3% phosphoric acid solution. 10 μL of the reaction is then spotted onto a P30 filtermat and washed three times for 5 min in 75 mM phosphoric acid and once in methanol prior to drying and scintillation counting.

### 4.3. Molecular Modelling

Compounds were docked into the FLT3 structure (PDB: 4RT7). Protein and ligand preparations were performed with Schrödinger’s tools with standard settings and Glide was used for docking and scoring. The 3 D X-ray protein structures of FLT3 wild-type as a complex with a ligand were obtained from the PDB (code: 4RT7) and prepared using the Protein Preparation Wizard of the Schrödinger Maestro program. All water molecules were removed from the structure and it was selected as a template. The structures of inhibitors were drawn using Chemdraw, and their 3 D conformation was generated using the Schrödinger LigPrep program with the OPLS 4 force field. Molecular docking of compound into the structure of FLT-3 wild-type (PDB code: 4RT7) were carried out using Schrodinger Glide (Version 12.7).

## Supplementary Material

Supplemental Material
