# Supplementary material for: Rational design and synthesis of 2-(1H-indazol-6-yl)-1H-benzo[d]imidazole derivatives as inhibitors targeting FMS-like tyrosine kinase 3 (FLT3) and its mutants
Source: J Enzyme Inhib Med Chem. 2022 Jan 23;37(1):472–86. doi: 10.1080/14756366.2021.2020772 (PMC8788362; doi:10.1080/14756366.2021.2020772)
Supplement: Supplemental Material [file IENZ_A_2020772_SM9894.pdf]

Supporting Information

Rational design and synthesis of 2-(1*H*-indazol-6-yl)-1*H*-benzo[d]imidazole derivatives. inhibitors targeting on FMS-like tyrosine kinase 3 (FLT3) and its mutants.

Daseul Im[a,b], Joonhong Jun[a,b], Jihyun Baek[a,b], Haejin Kim[a,b], Dahyun Kang[a,b], Hyunah Bae[a,b], Hyunwook Cho[a,b], Jung-Mi Hah*

[a]*Department of Pharmacy, College of Pharmacy, Hanyang University, Ansan, Korea;*

[b]*Institute of Pharmaceutical Science and Technology, Center for Proteinopathy, Hanyang University, Ansan, Korea*

**Correspondence: Jung-Mi Hah*
Email: jhah@hanyang.ac.kr; Tel.: +82-31-400-5803, Hanyang University, 55 Hanyandaehak-ro Sannok-gu, Ansan, Gyeonggi-do, 15588 Korea

Contents:

1. Copies of ^{1}H and ^{13}C NMR spectrum of selected compounds 2

2. Percentages of enzymatic inhibition exerted by **8r** toward selected protein kinases 16

3. References 18

1. Copies of ^{1}H and ^{13}C NMR spectrum of selected compounds

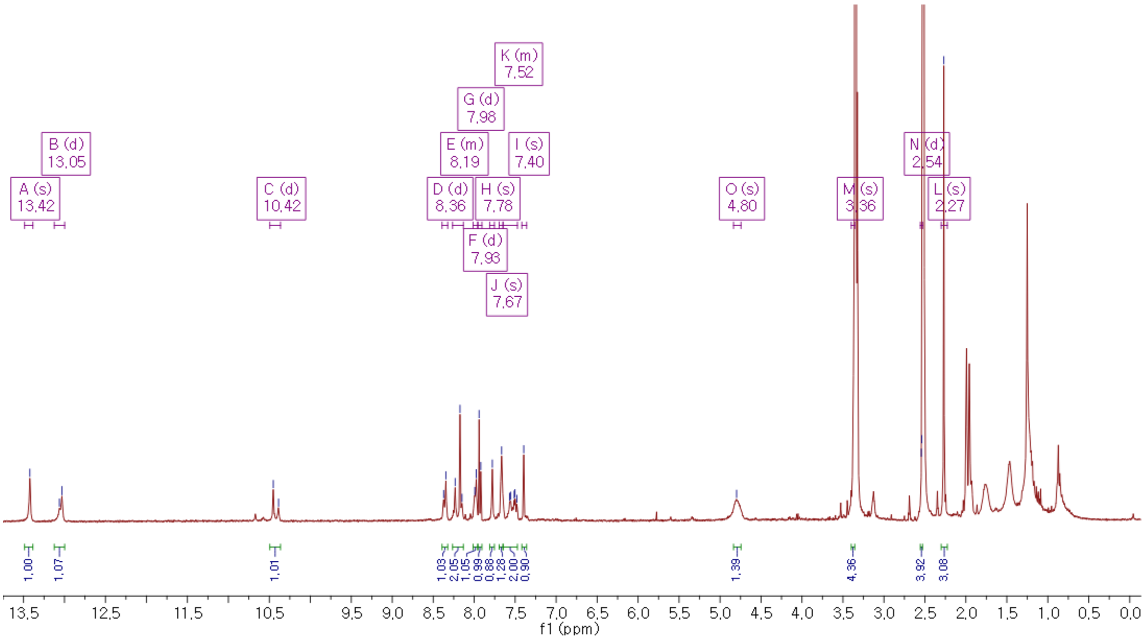

Figure S1. ^{1}**H NMR** spectrum of compound **8a**

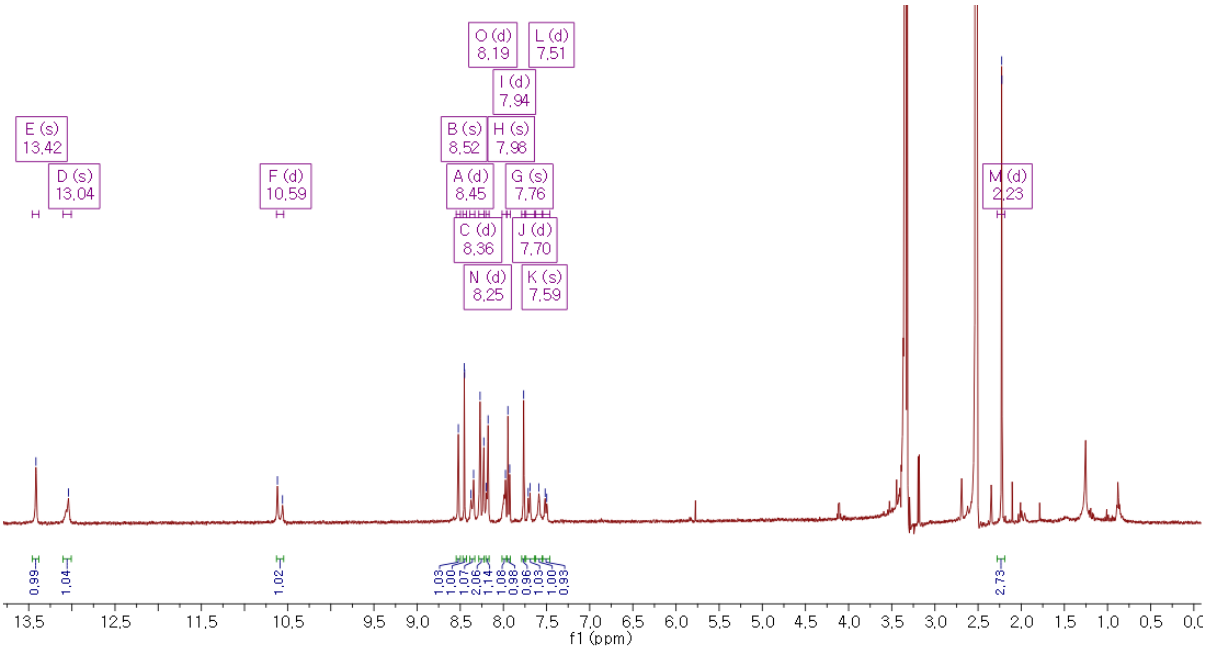

Figure S2. ^{1}**H NMR** spectrum of compound **8b**

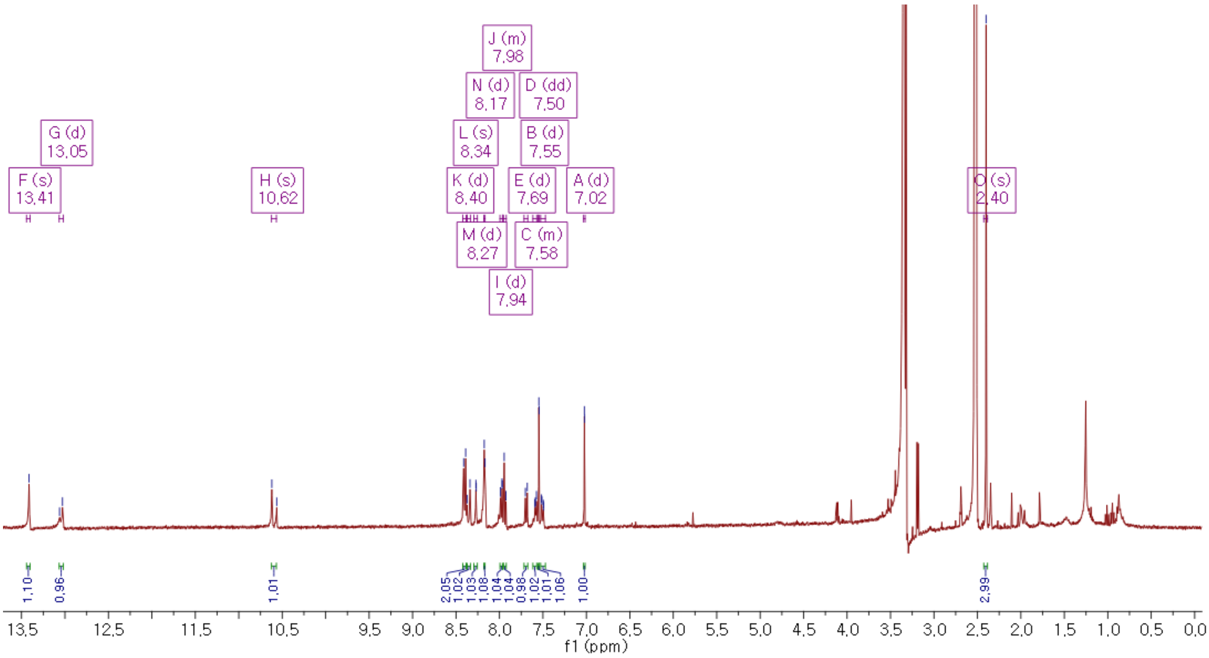

Figure S3. [1]**H NMR** spectrum of compound **8c**

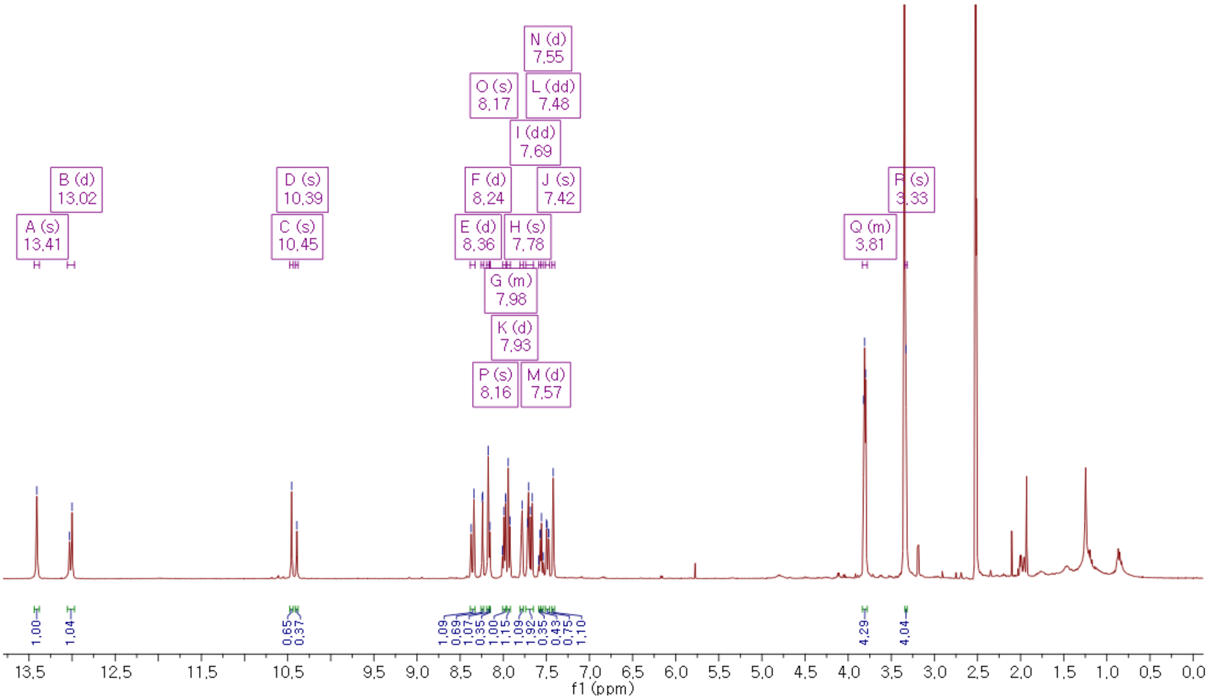

Figure S4. [1]**H NMR** spectrum of compound **8d**

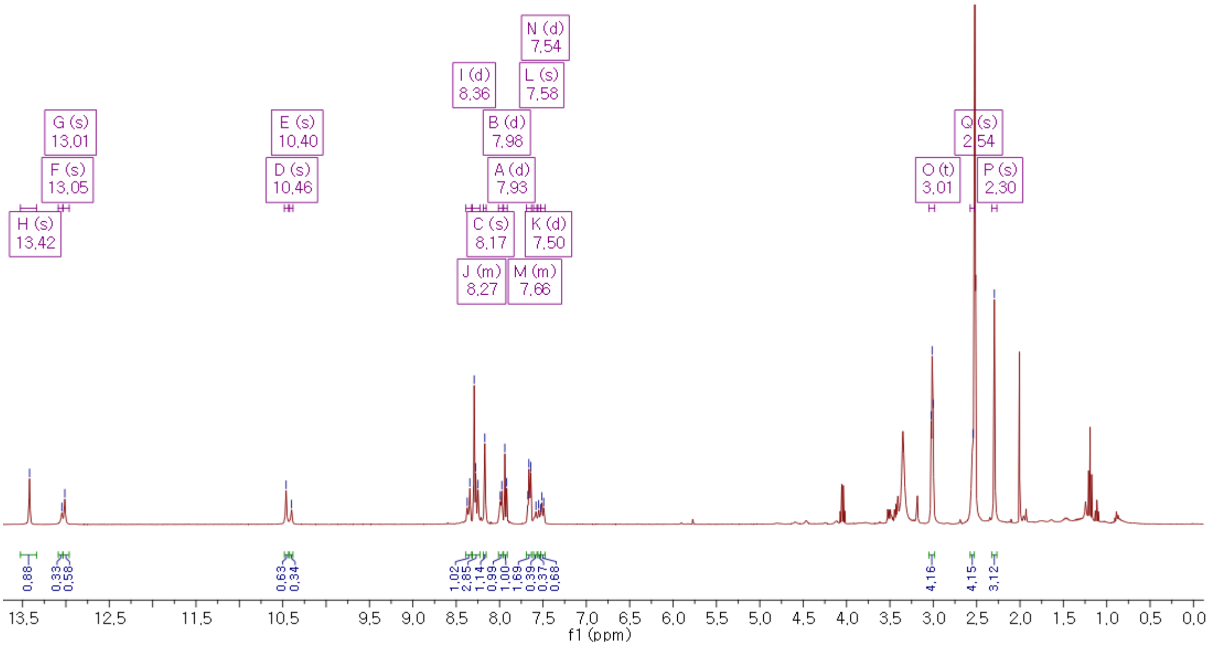

Figure S5. [1]**H NMR** spectrum of compound **8e**

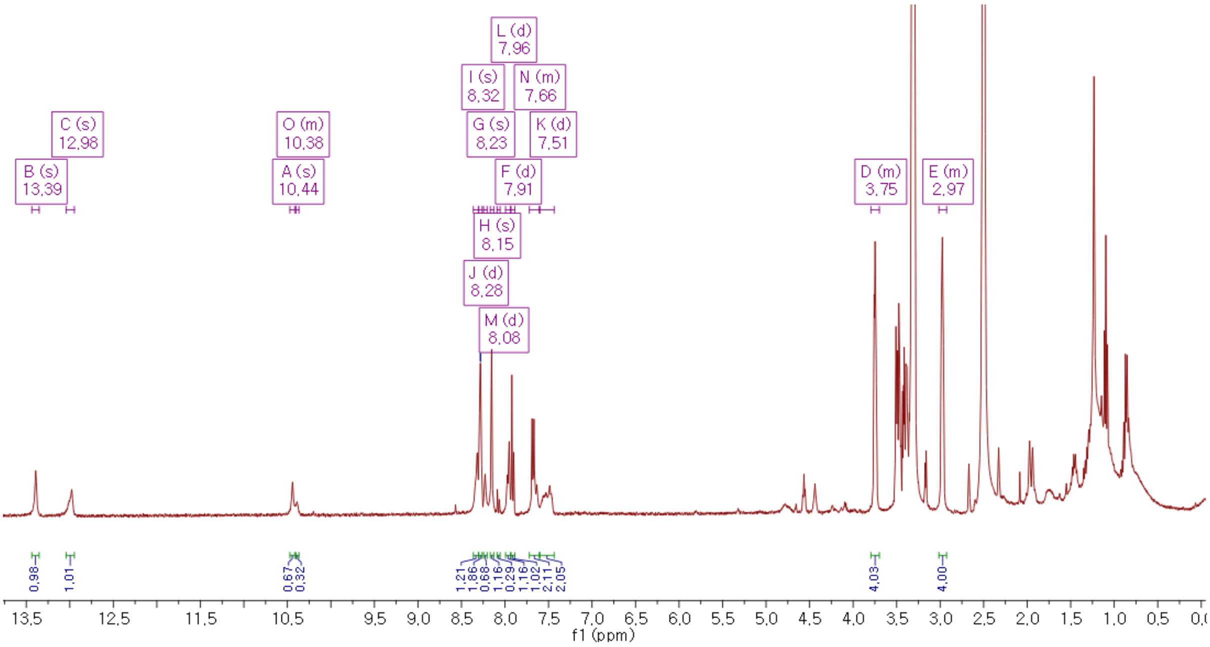

Figure S6. [1]**H NMR** spectrum of compound **8f**

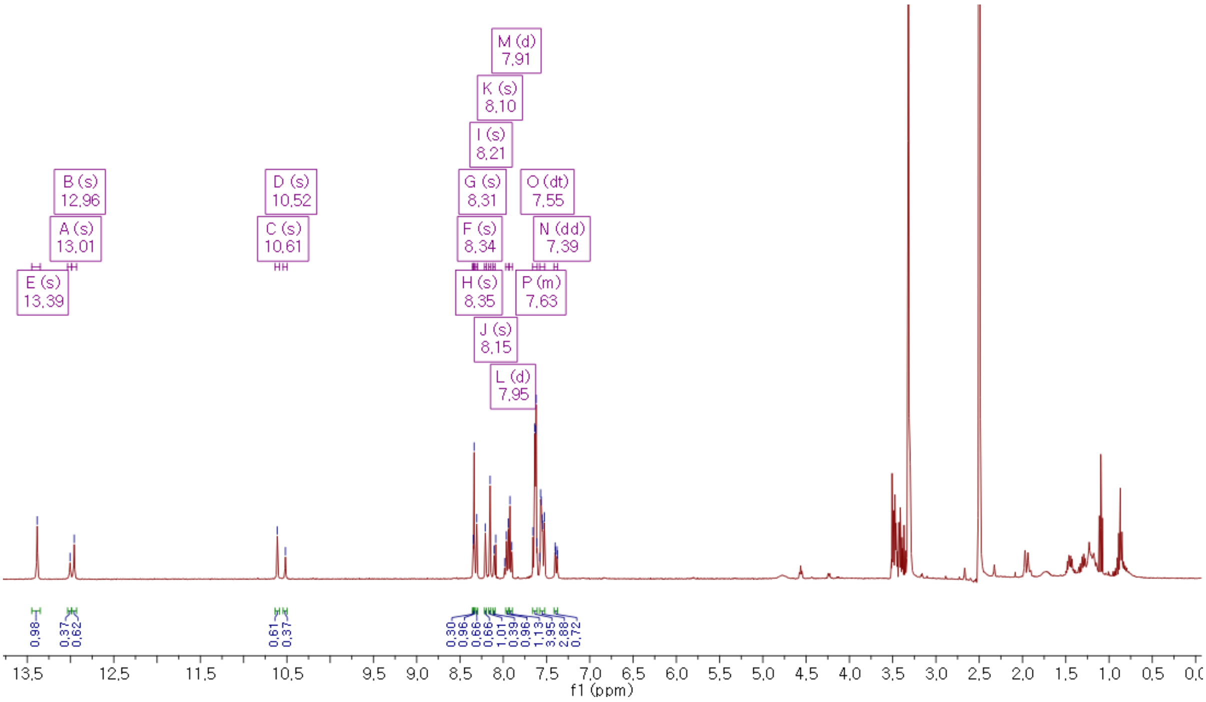

Figure S7. [1]**H NMR** spectrum of compound **8g**

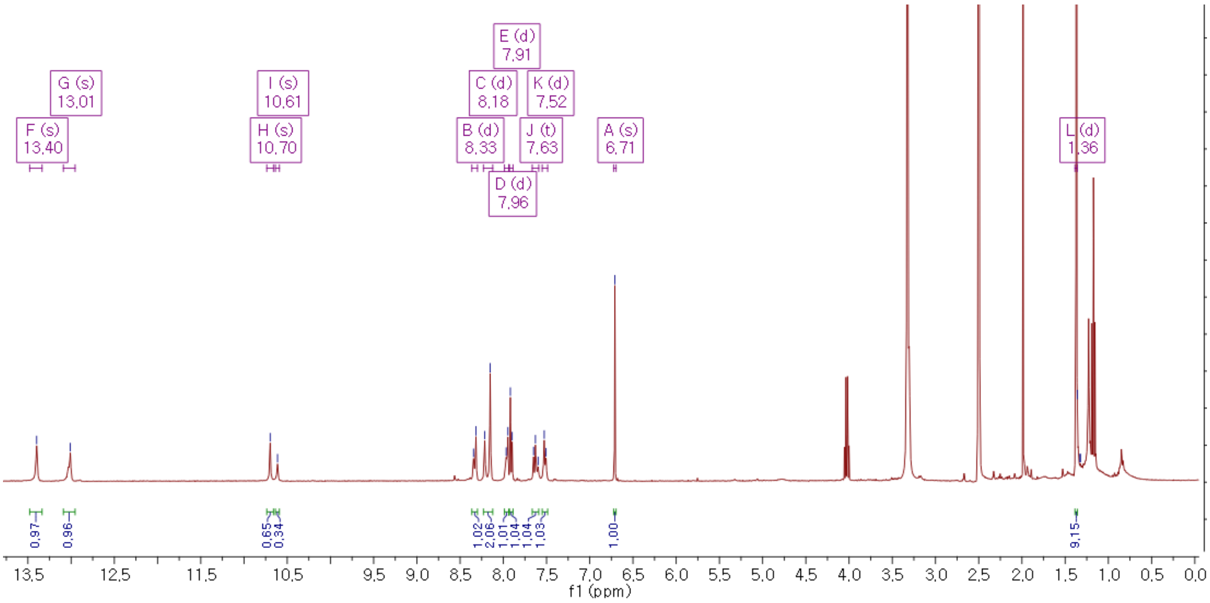

Figure S8. [1]**H NMR** spectrum of compound **8h**

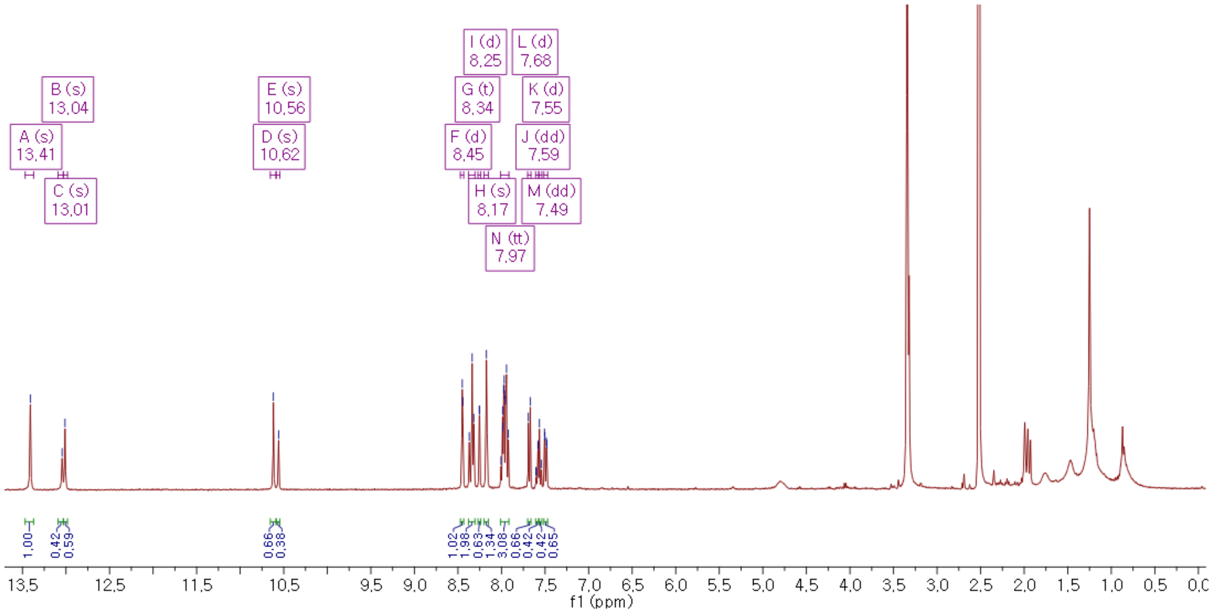

Figure S9. [1]**H NMR** spectrum of compound **8i**

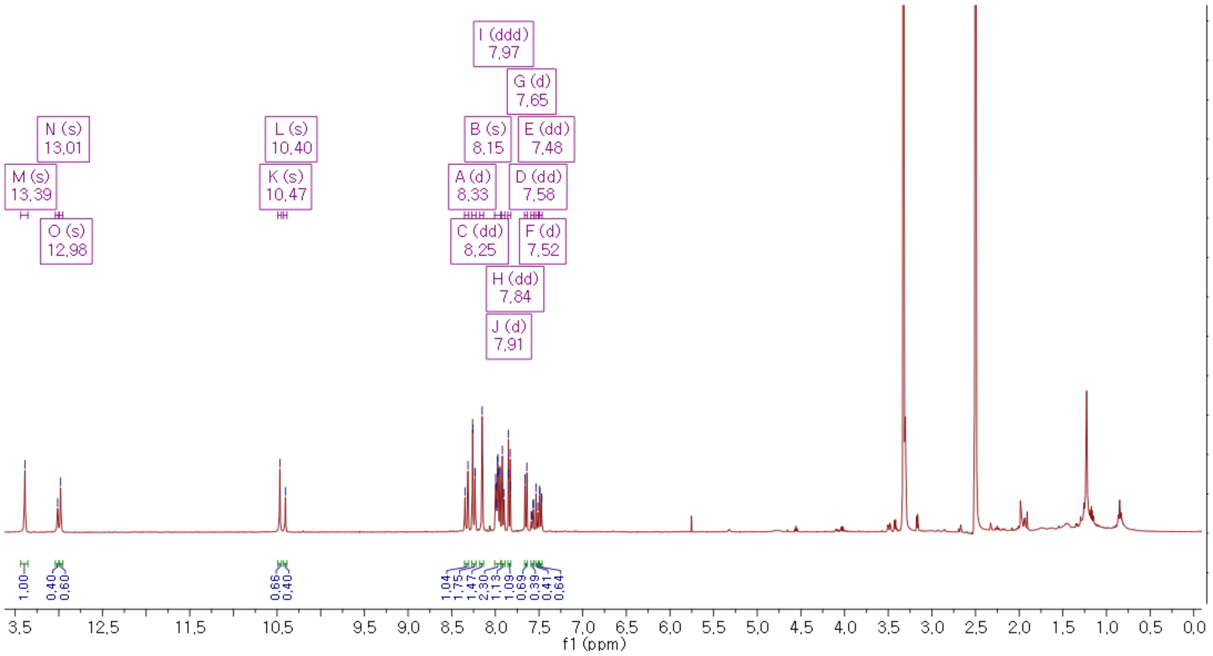

Figure S10. [1]**H NMR** spectrum of compound **8j**

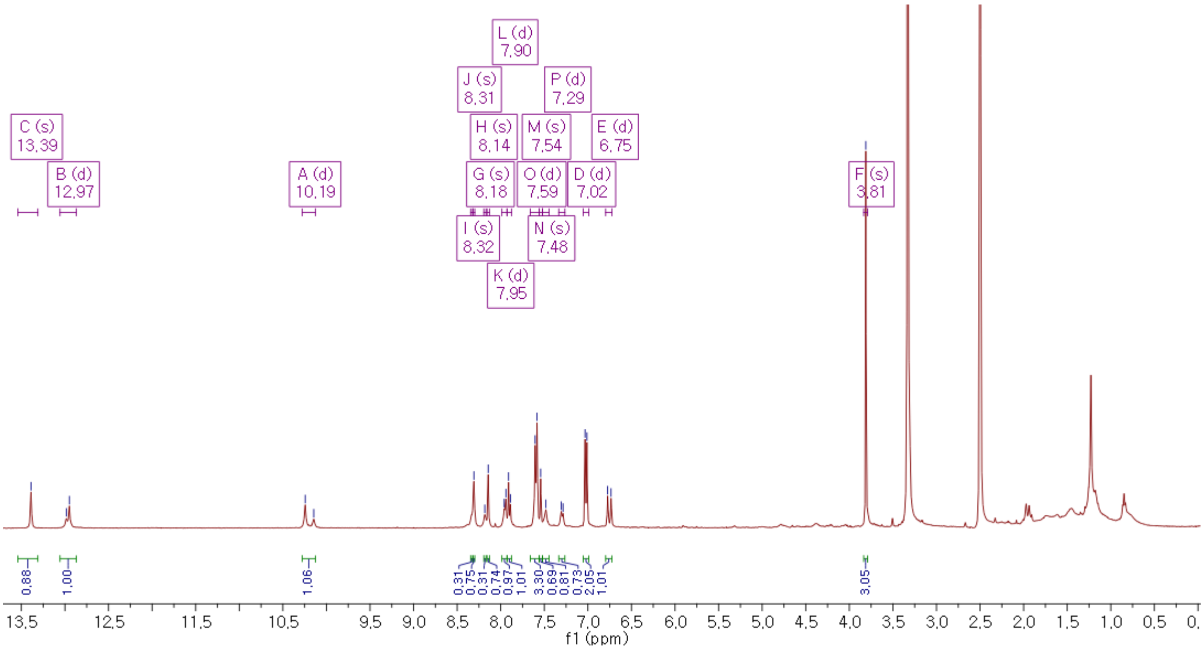

Figure S11. [1]**H NMR** spectrum of compound **8k**

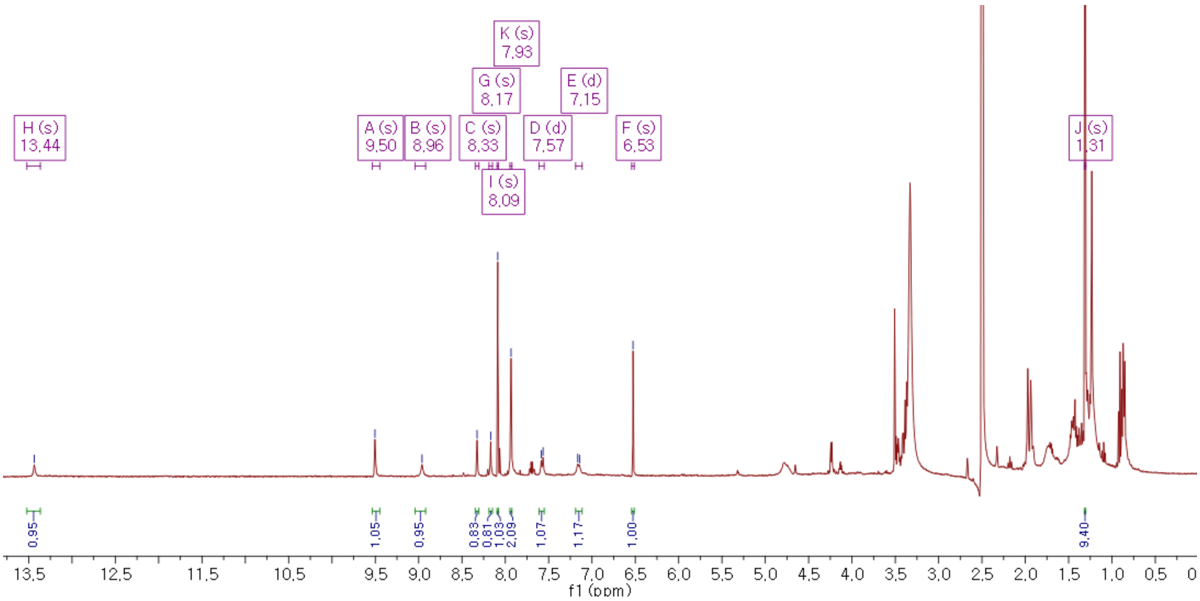

Figure S12. [1]**H NMR** spectrum of compound **8l**

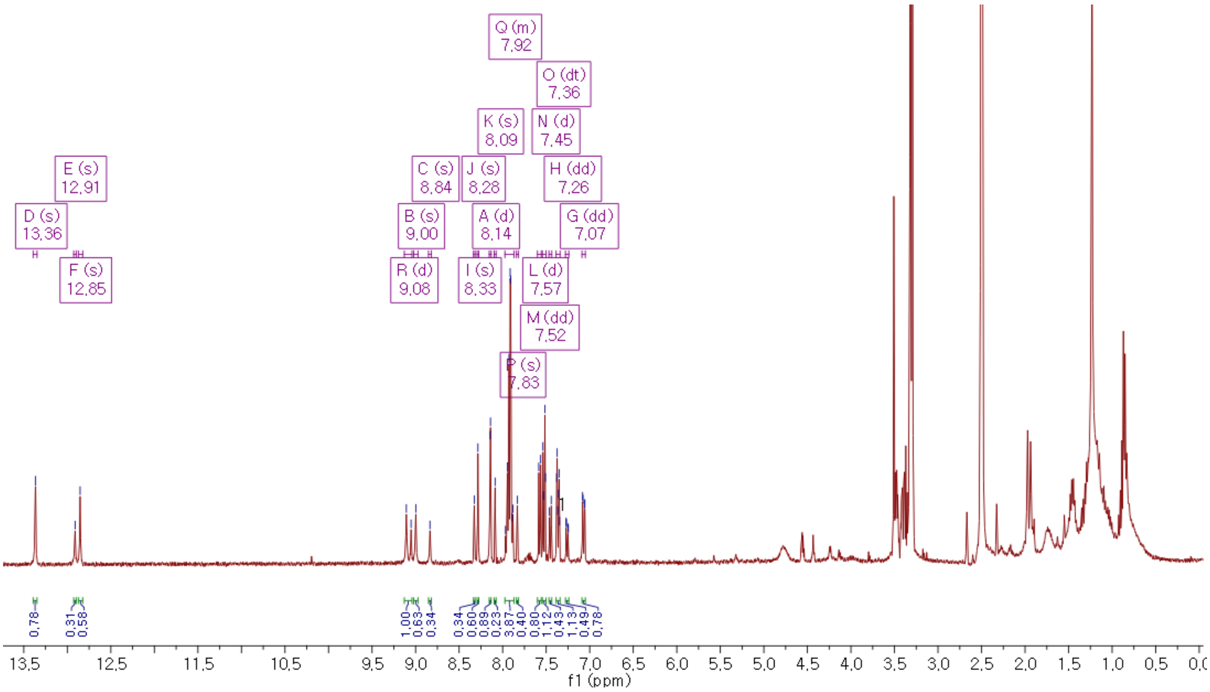

Figure S13. [1]**H NMR** spectrum of compound **8m**

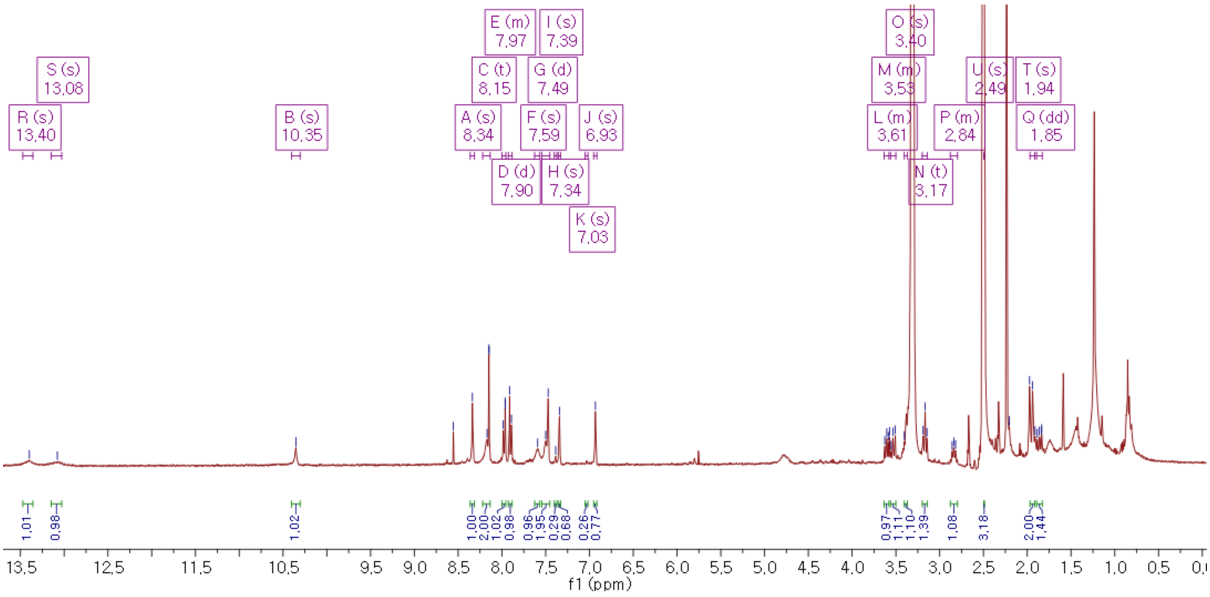

Figure S14. [1]**H NMR** spectrum of compound **8n**

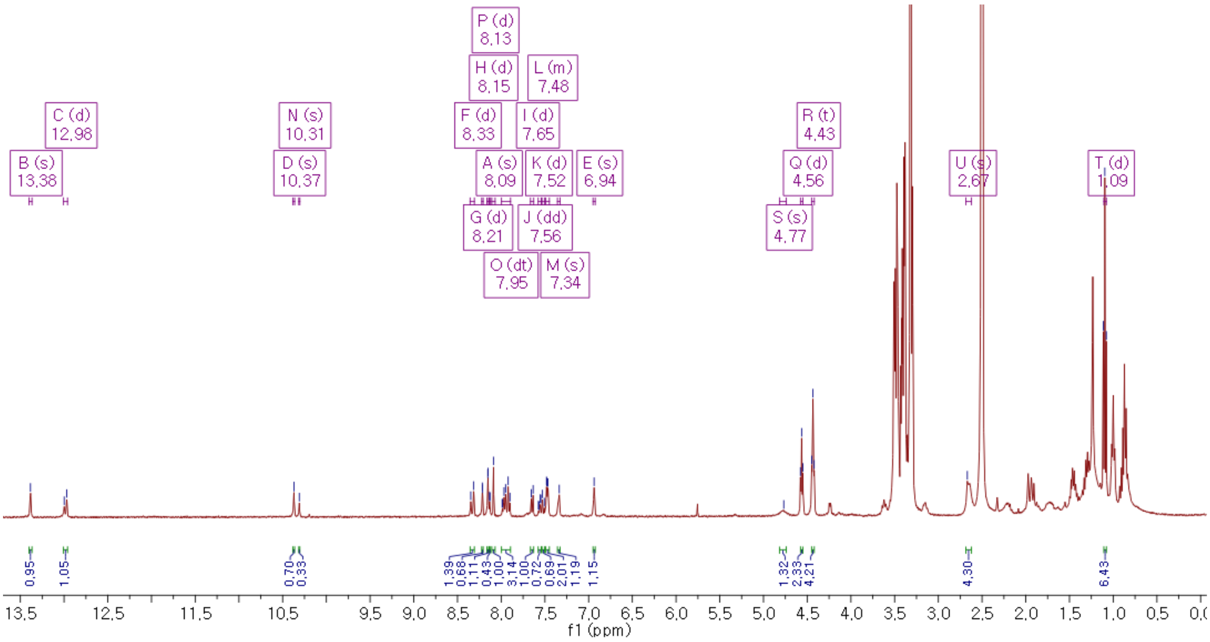

Figure S15. **¹H NMR** spectrum of compound **8o**

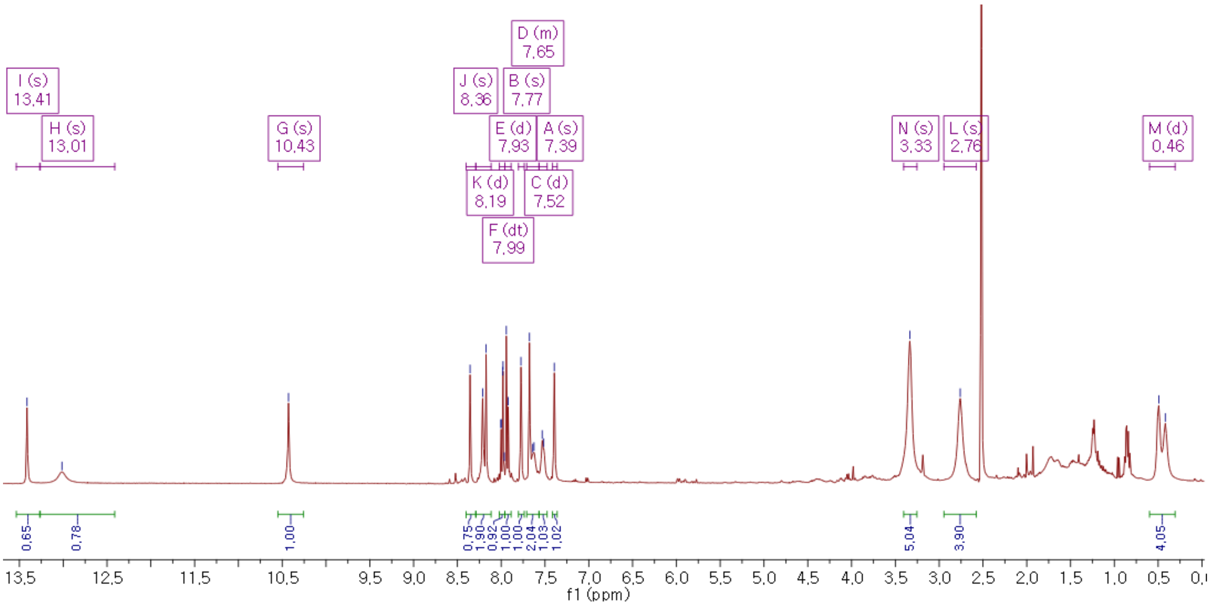

Figure S16. **¹H NMR** spectrum of compound **8p**

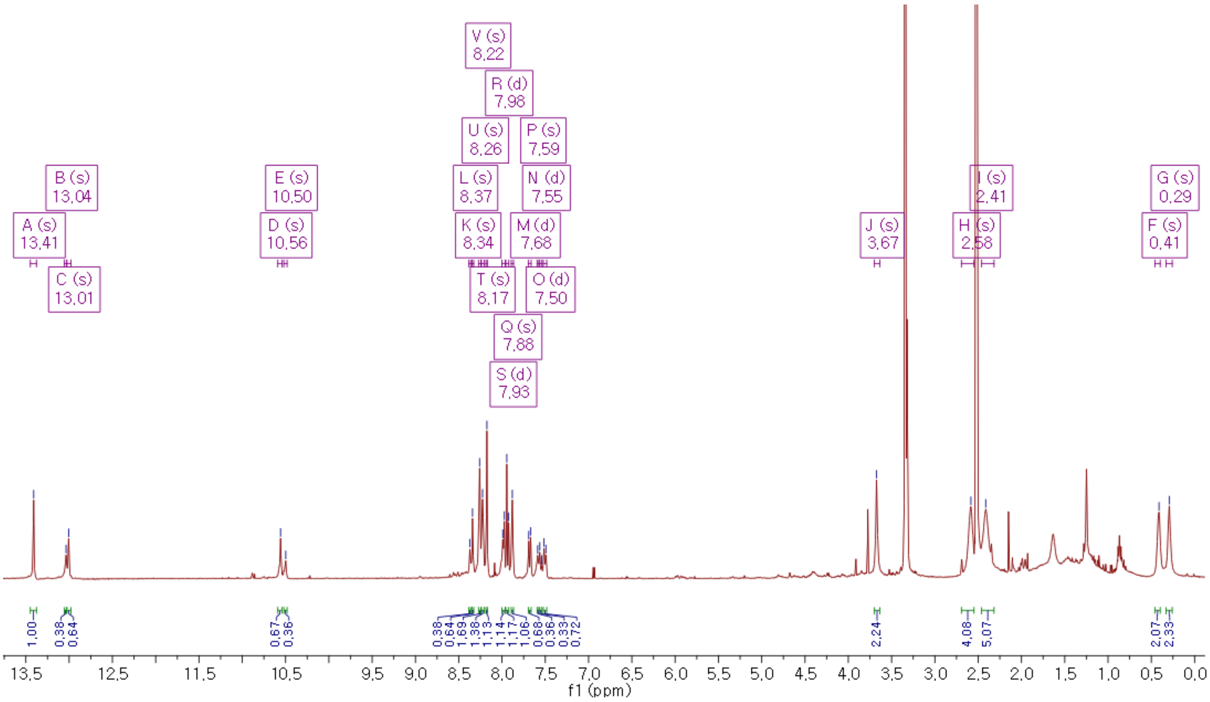

Figure S17. [1]**H NMR** spectrum of compound **8q**

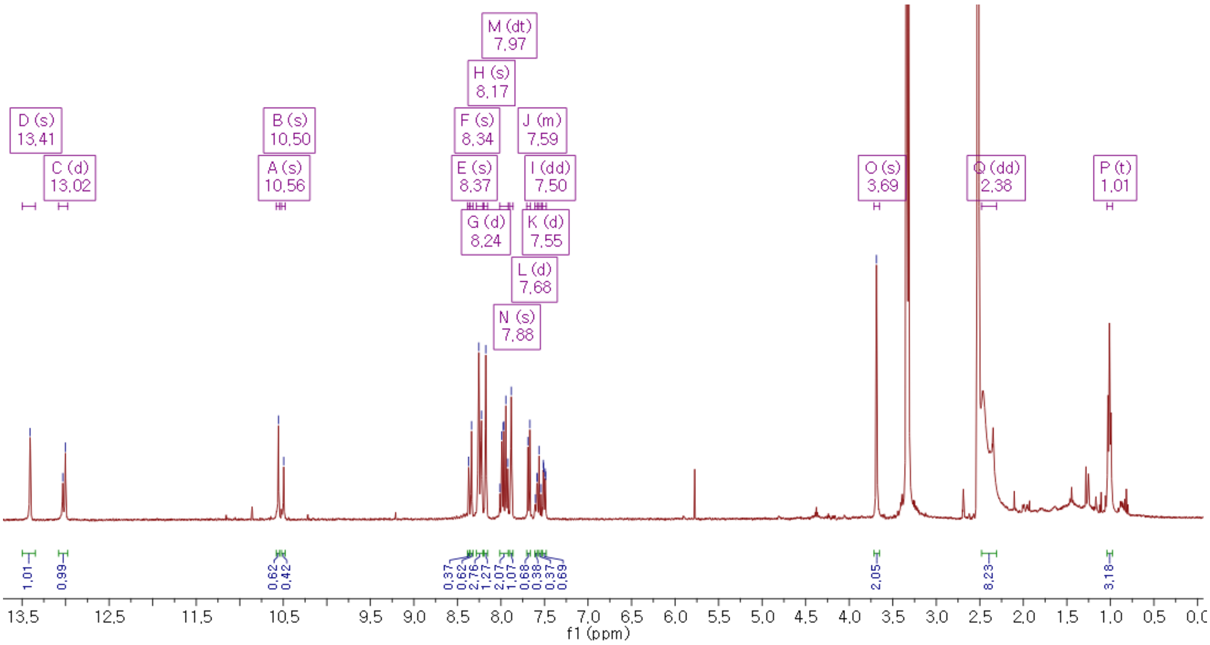

Figure S18A. [1]**H NMR** spectrum of compound **8r**

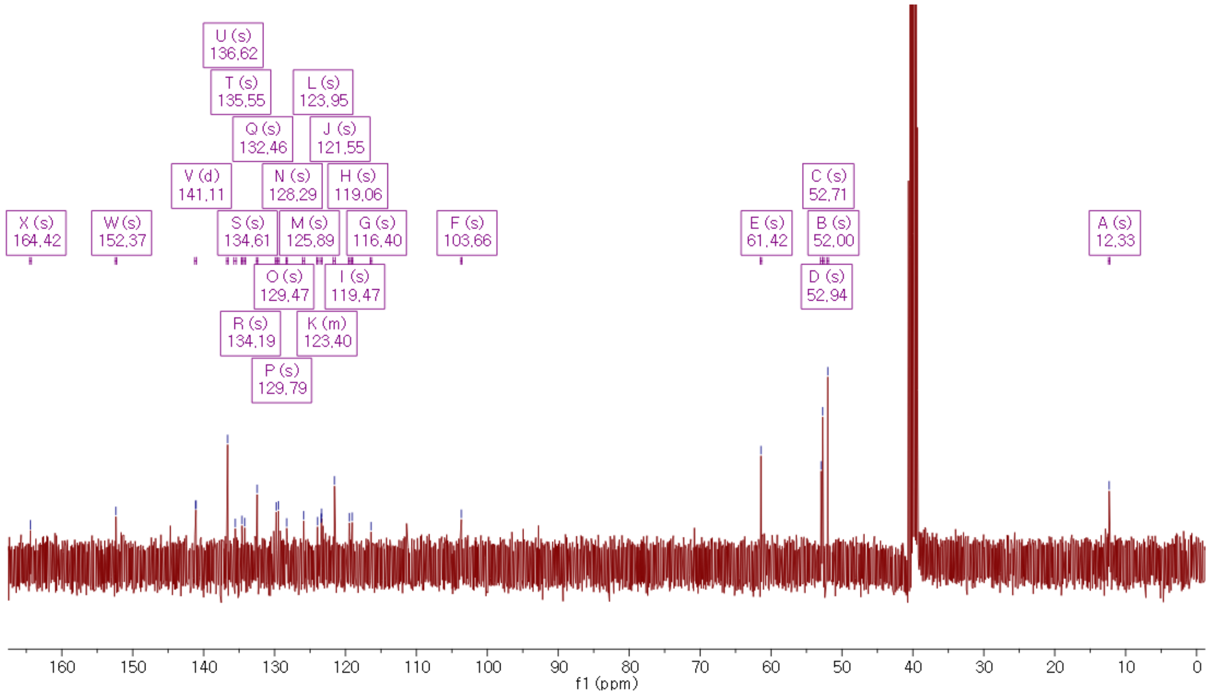

Figure S18B. ^{13}**C NMR** spectrum of compound **8r**

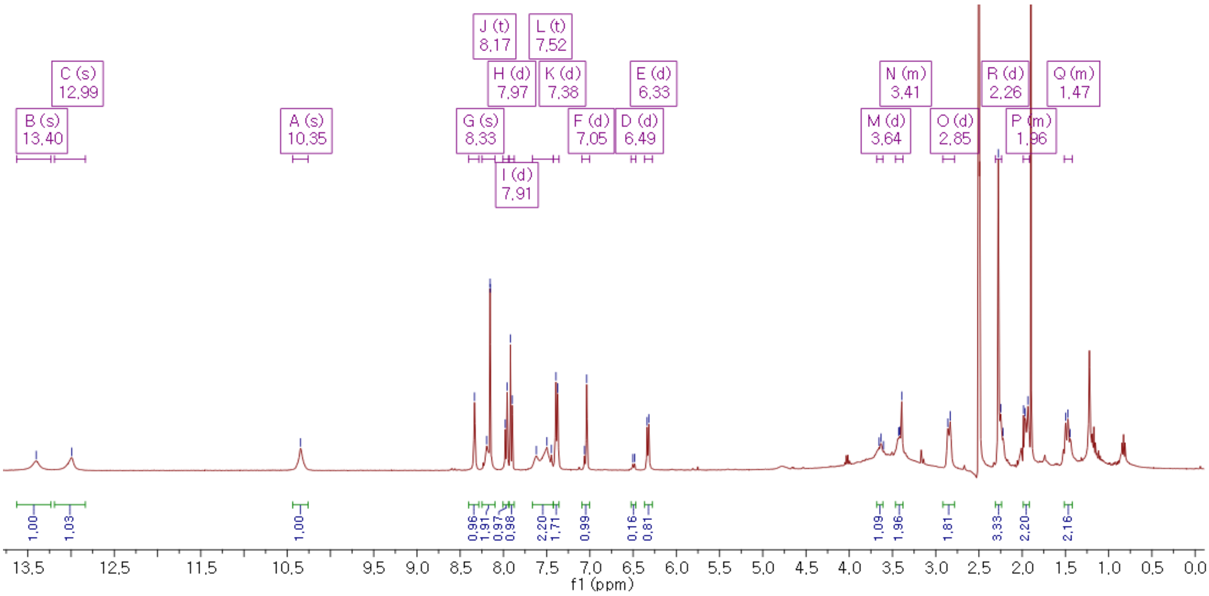

Figure S19. ^{1}**H NMR** spectrum of compound **8s**

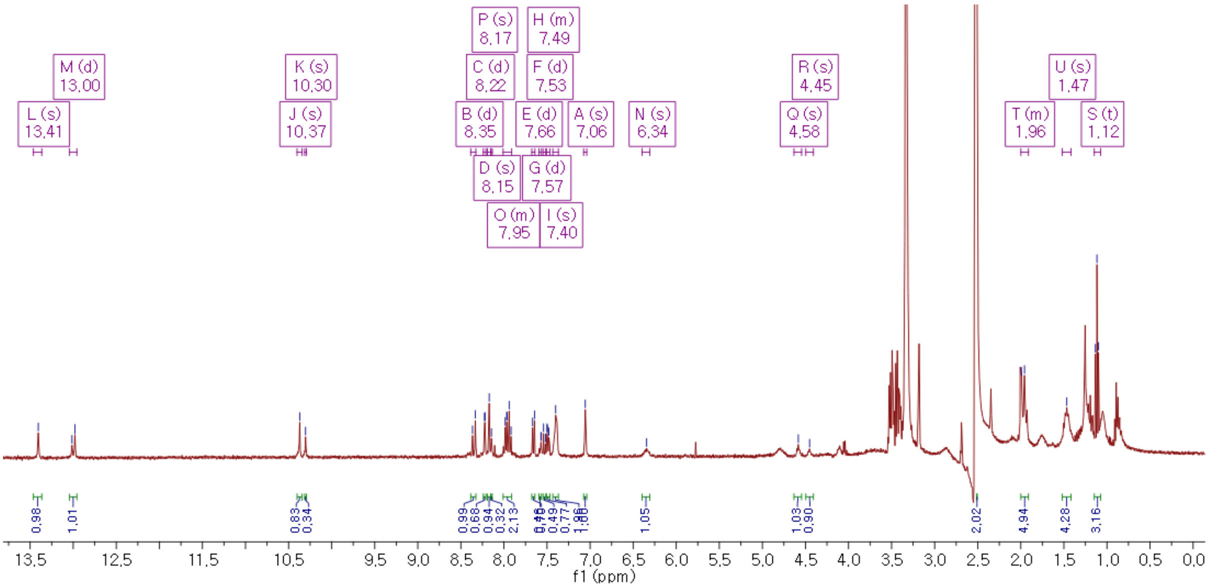

Figure S20. ^{1}**H NMR** spectrum of compound **8t**

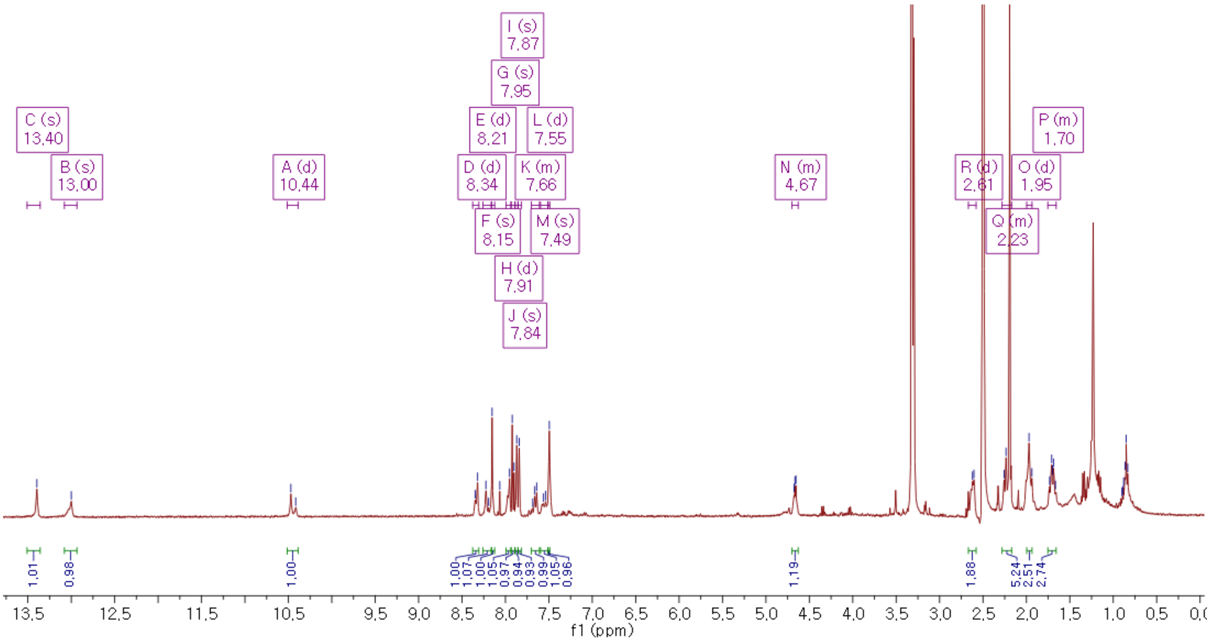

Figure S21. ^{1}**H NMR** spectrum of compound **8u**

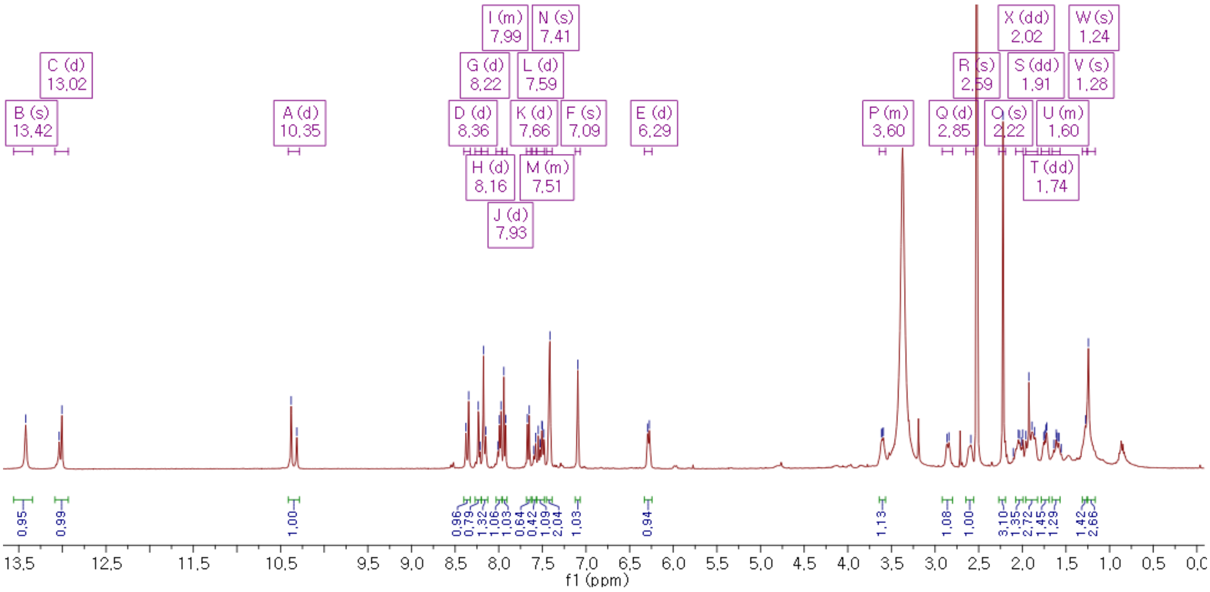

Figure S22. [1]**H NMR** spectrum of compound **8v**

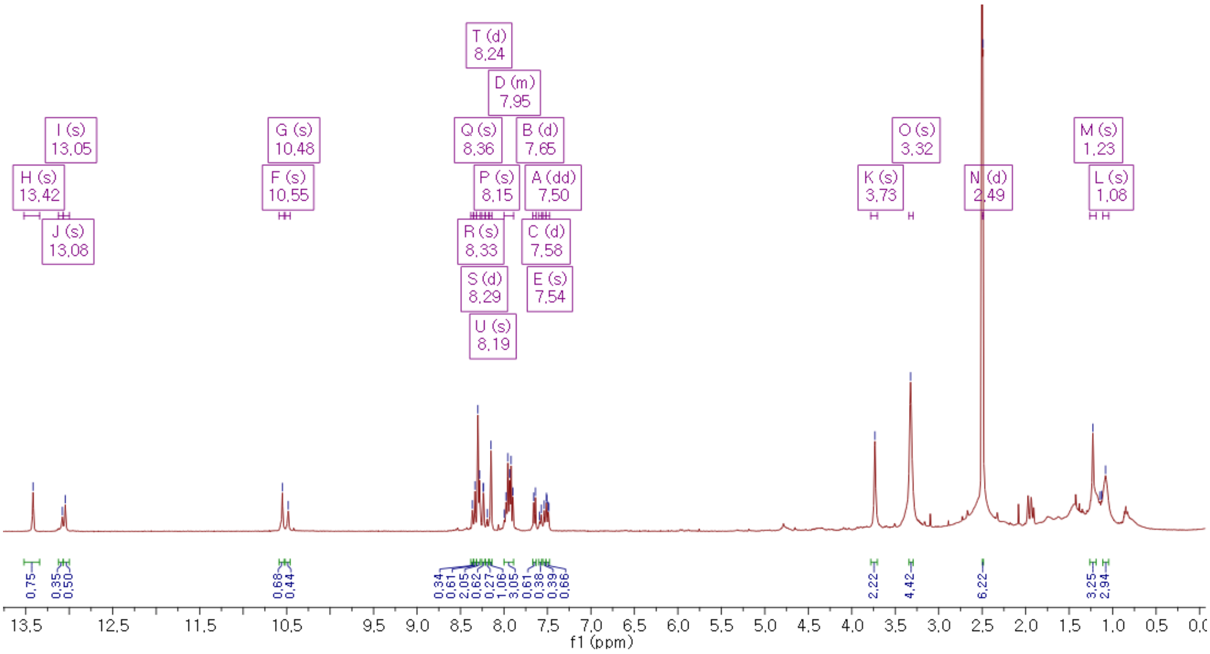

Figure S23. [1]**H NMR** spectrum of compound **8w**

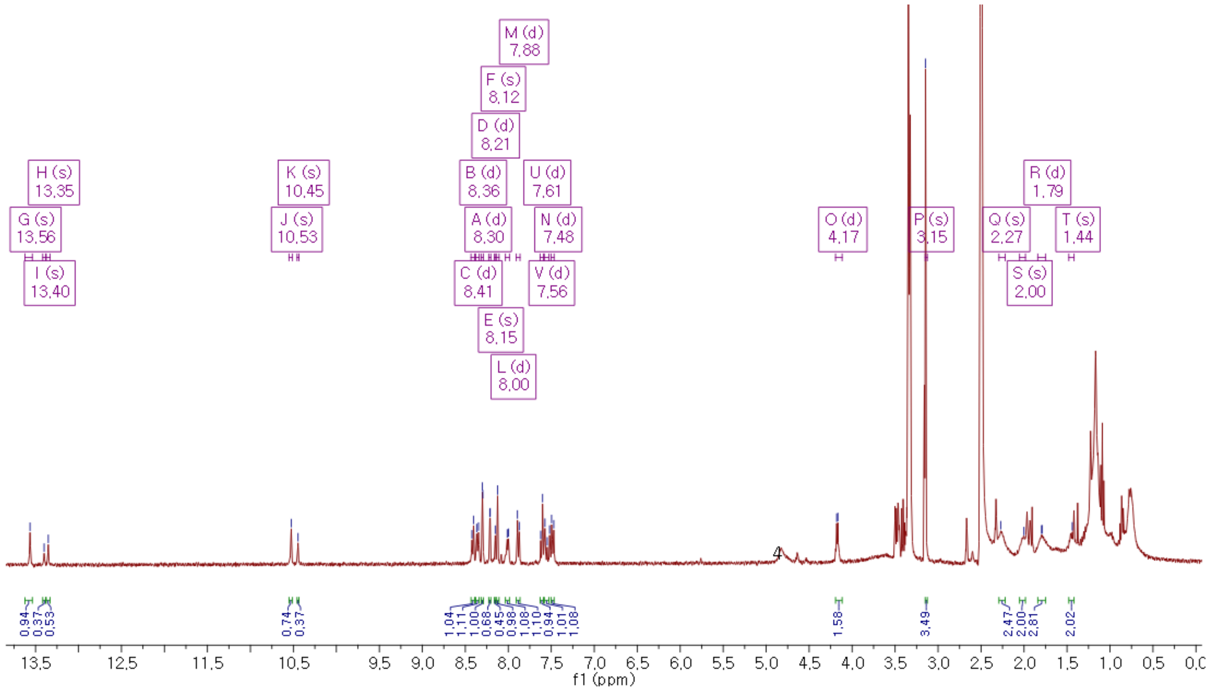

Figure S24. [1]**H NMR** spectrum of compound **8x**

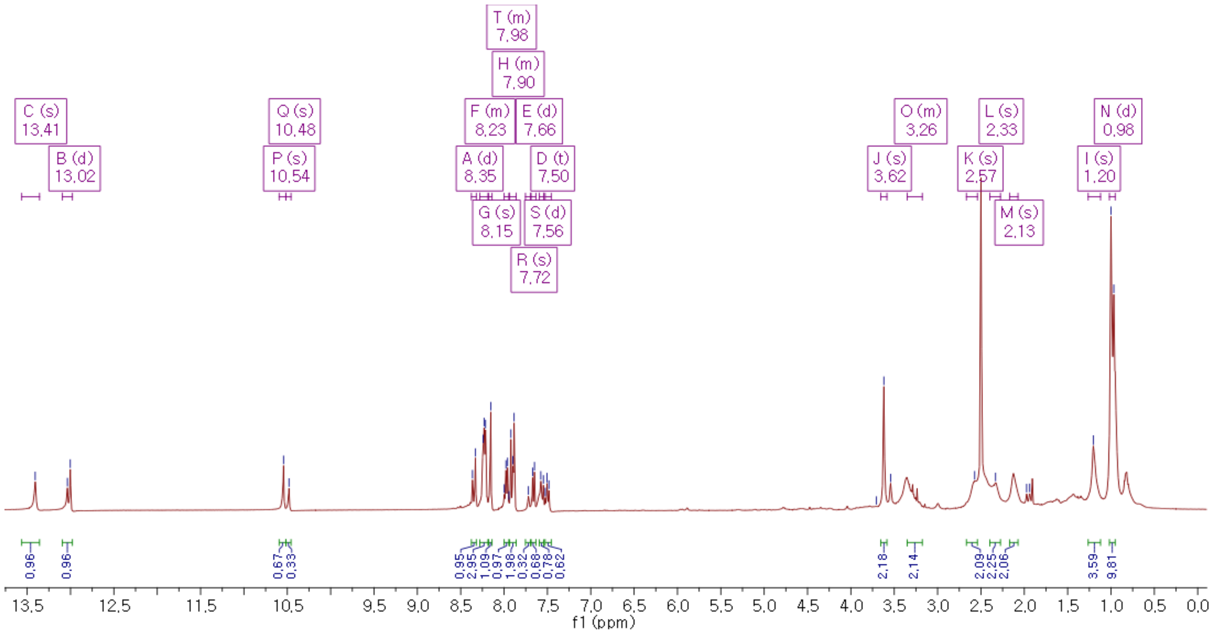

Figure S25. [1]**H NMR** spectrum of compound **8y**

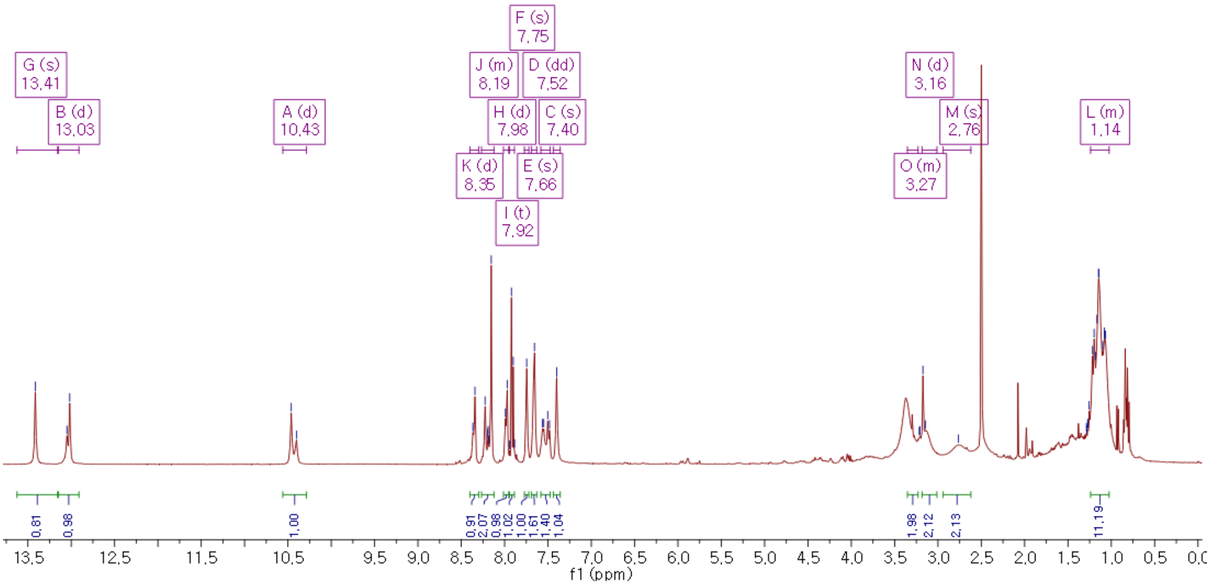

Figure S26. [1]**H NMR** spectrum of compound **8z**

2. Percentages of enzymatic inhibition exerted by 8r toward selected protein kinases

Table S1. Percentages of enzymatic inhibition exerted by **8r** (1 μM) toward **42** selected protein kinases.

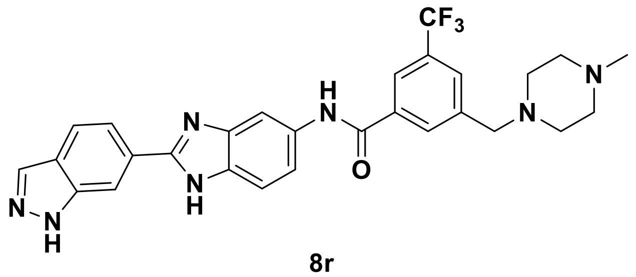

8r

Kinase	% Inhibition	Staurosporine IC_{50} (nM)
ABL1	18.4	29.1
AKT1	3.09	3.21
ALK	7.50	1.76
Aurora A	7.47	2.25
AXL	1.88	6.42
AXL (R499C)	6.64	1.32
BRAF (V599E)	1.00	13.1[a]
BTK	17.5	12.8
c-Kit	19.0	1.05
c-MER	7.30	9.65
c-MET	6.53	26.1
c-Src	1.94	1.59
CAMKK1	32.5	67.6
CDK4/cyclin D1	8.26	7.59
EGFR	0.274	39.0
ERK1	0	15.0[b]
FGFR3	0	7.63
FLT1/VEGFR1	6.00	4.32
FLT3 (wild)	100	7.16
FLT3 (F594_R595insR)	92.0	2.34
FLT3 (F594_R595insREY)	92.0	2.02
FLT3 (ITD)-NPOS	88.0	1.66
FLT3 (ITD)-W51	93.9	1.95
FLT3 (R595_E596insEY)	93.9	1.72
FLT3 (Y591_V592insVDFREYEYD)	90.9	2.02
FLT3-ITD	88.0	1.86
FMS	0.531	3.10
FYN	17.96	<1.00

GSK3b	7.25	8.15
IGF1R	6.145	14.8
JAK3	6.42	<1.00
KDR/VEGFR2	7.12	11.4
LCK	0	3.56
LYN	7.40	<1.00
MEK1	5.69	56.0
PKA	7.34	1.0
PLK1	5.39	143
RON/MST1R	6.99	71.2
ROS/ROS1	7.40	<1.00
SYK	6.01	<1.00
TRKC	48.3	<1.00
TYRO3/SKY	9.76	1.32

[a] Data of GW5074[1]

[b] Data of SCH772984[2,3]

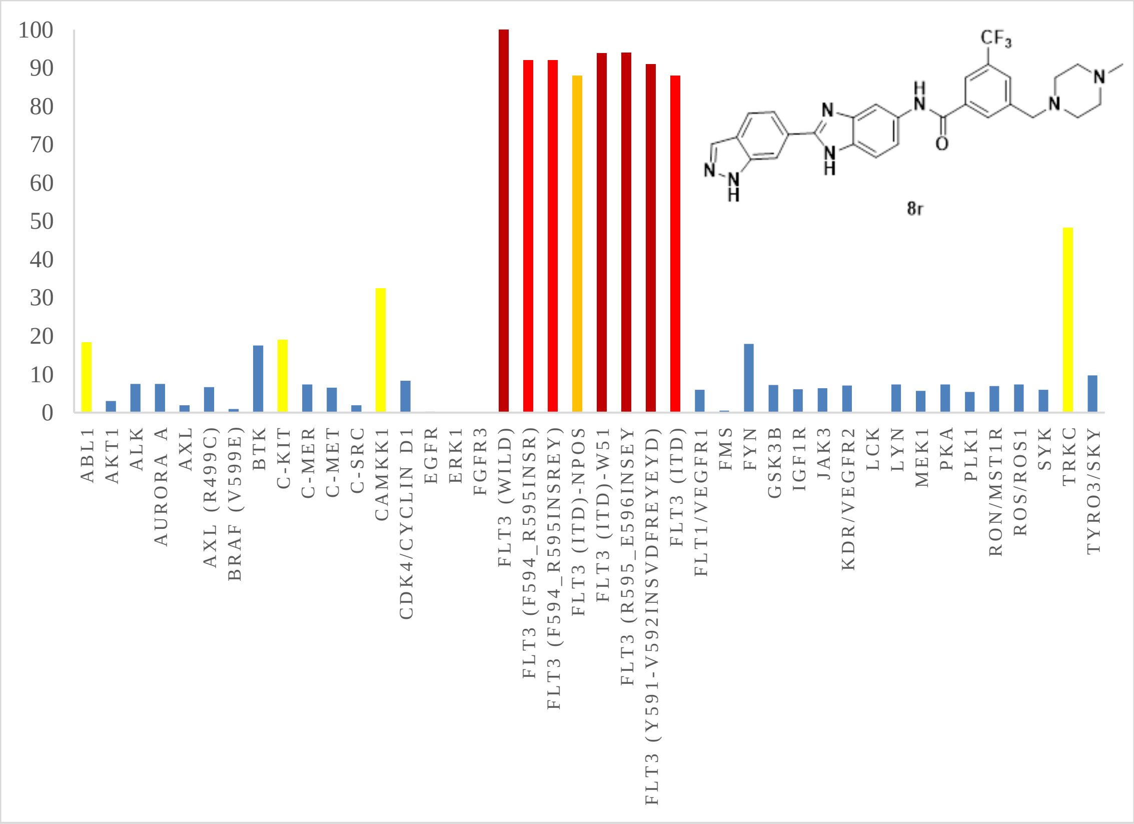

Figure S27. Percentages of enzymatic inhibition exerted by **8r** toward 42 selected protein kinases

3. References

1. K. Lackey, M. Cory, R. Davis, S.V. Frye, P.A. Harris, R.N. Hunter, D.K. Jung, O.B. McDonald, R.W. McNutt, M.R. Peel, R.D. Rutkowske, J.M. Veal, E.R. Wood, The discovery of potent cRaf1 kinase inhibitors, Bioorg. Med. Chem. Lett. 2000;10(10): 223-226.
2. Seger R, Krebs EG. The MAPK signaling cascade. *FASEB. J.* 1995;9(9):726-735.
3. Morris EJ, Jha S, Restaino CR, Dayananth P, Zhu H, Cooper A, et al. Discovery of a novel ERK inhibitor with activity in models of acquired resistance to BRAF and MEK inhibitors. *Cancer Discov.* 2013;3(7):742–750.
